# Modelling the simultaneous encoding/serial experience theory of the perceptual moment: a blink of meta-experience

**DOI:** 10.1093/nc/niac003

**Published:** 2022-03-02

**Authors:** Howard Bowman, William Jones, Hannah Pincham, Steve Fleming, Axel Cleeremans, Murray Smith

**Affiliations:** School of Computing, University of Kent, Canterbury, Kent CT2 7NF, UK; Department of Psychology, University of Birmingham, Birmingham B15 2TT, UK; School of Computing, University of Kent, Canterbury, Kent CT2 7NF, UK; School of Psychiatry, Faculty of Medicine, University of New South Wales, Kensington, NSW 2052, Australia; Wellcome Centre for Human Neuroimaging, and Max Planck University College London Centre for Computational Psychiatry and Ageing Research, 12, Queen Square, University College London, London WC1N 3AR, UK; Consciousness, Cognition & Computation Group, Center for Research in Cognition & Neuroscience, ULB Neuroscience Institute, Université libre de Bruxelles, 50 ave. F.-D. Roosevelt CP191, Brussels B-1050, Belgium; School of Arts, University of Kent, Jarman Building, Canterbury, Kent CT2 7UG, UK

**Keywords:** meta-cognition, perceptual moment, meta-experience, simultaneous encoding/ serial experience, attentional blink, blink of bits, experiential blink, meta-experiential blink

## Abstract

One way to understand a system is to explore how its behaviour degrades when it is overloaded. This approach can be applied to understanding conscious perception by presenting stimuli in rapid succession in the ‘same’ perceptual event/moment. In previous work, we have identified a striking dissociation during the perceptual moment, between what is encoded into working memory [Lag-1 sparing in the attentional blink (AB)] and what is consciously perceived (Lag-1 impairing in the experiential blink). This paper links this dissociation to what, taking inspiration from the metacognition literature, could be called meta-experience; i.e. how the ability to track and comment on one’s visual experience with subjectivity ratings reflects objective performance. Specifically, we provide evidence that the information (in bits) associated with an encoding into working memory decouples from the experiential reflection upon that perceptual/encoding event and that this decoupling is largest when there is the greatest perceptual overload. This is the meta-experiential blink. Meta-experiential self-observation is common to many computational models, including connectionist interpretations of consciousness, Bayesian observers and the readout-enhanced simultaneous type/serial token (reSTST) model. We assess how our meta-experiential blink data could be modelled using the concept of self-observation, providing model fits to behavioural and electroencephalogram responses in the reSTST model. We discuss the implications of our computational modelling of parallel encoding but serial experience for theories of conscious perception. Specifically, we (i) inform theories of Lag-1 sparing during the AB and (ii) consider the implications for the global workspace theory of conscious perception and higher-order theories of consciousness.

## Introduction

The modelling we present here takes inspiration from higher-order thought (HOT) theories of consciousness (Rosenthal 2005; Lau and Rosenthal 2011), according to which a mental state (whether perceptual or cognitive) is conscious if one is aware of being in that mental state. While this might sound circular, it merely suggests that first-order mental states become conscious mental states when there is a ‘higher-order thought’—a meta-representation—indicating the existence of the target first-order state to the agent. Crucially, the higher-order state does not itself need to be conscious. Thus, unconscious higher-order mental states render their target first-order states conscious. This suggests that conscious experience depends on the operation of a meta level that minimally takes the form of an internal observer. Beyond HOT theory itself (Rosenthal 2005), five extant theories of consciousness broadly espouse such a view: (i) Higher-Order Representation Of a Representation theory (Brown 2015; Brown *et al.* 2019), (ii) Perceptual Reality Monitoring theory (Lau 2019), (iii) the Higher-Order State Space model (HOSS, Fleming 2020), (iv) the Self-Organizing Metarepresentational Account (Cleeremans *et al.* 2020) and (v) the readout-enhanced simultaneous type/serial token (reSTST) model (Jones *et al.* 2020). We elaborate on the latter of these in this paper.

The key to constraining and validating these theories is to understand the subtle interplay between objective performance and subjective experience. In this paper, this becomes the interplay between working memory encoding and conscious visual experience, when multiple (target) stimuli are presented in rapid succession in the ‘same’ perceptual event/moment. Examples of such rapid presentation of stimuli include Lag-1 (second target, T2, immediately following first target, T1) in the attentional blink (AB) (Hommel and Akyürek 2005; Wyble *et al.* 2009), temporal conjunction errors (features of one target reported with another) (Botella *et al.* 2001; Chennu *et al.* 2011), the perceptual moment itself (the time period within which two stimuli can be experienced as a single combined percept) (Di Lollo and Wilson 1978) and postdictive effects in general (Sergent 2018; Herzog *et al.* 2020).

**Figure 1.**
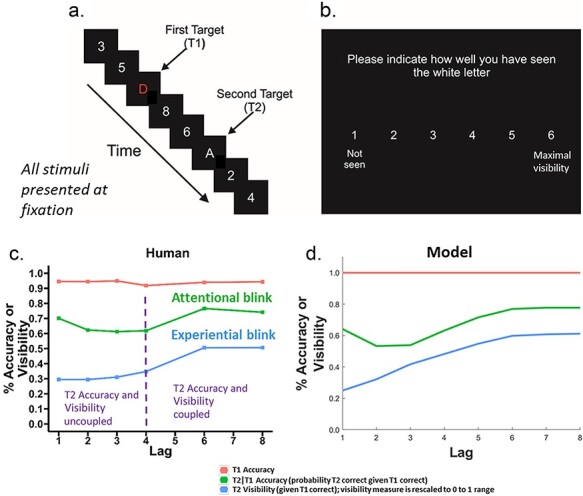
Blink experiment and behavioural findings for humans and model: (a) Rapid serial visual presentation (RSVP) stream, in which T1 and T2 were letters presented between white digit distractors. T1 was always a red letter and T2 was a white letter. In this figure, T2 appears at Lag 3, i.e. with two intervening distractors. Items appeared for 90 ms duration. (b) Phrasing and screen layout of subjective visibility question, which asked about the white letter and therefore referred to T2 and not T1. (c) Serial-position curves of T1 Accuracy, T2|T1 Accuracy and T2 subjective visibility from human data, and (d) simulated results for the noisy reSTST model. T2 accuracy and visibility are strongly coupled from lags 4 upwards (i.e. there would be no interaction between measure and lag) but uncoupled as lag reduces from 3 downwards (i.e. a measure by lag interaction would be present). Note: the model fits shown in (d) are slightly different to those in Jones *et al*. (2020). This is because we are using a noisy version of the reSTST model here, i.e. which models noise—see section ‘Materials and Methods’, subsection ‘Readout-enhanced STST model’ for details. Panels a and b are reproduced from Pincham *et al*. (2016), with permission, copyright Elsevier.

Here, we focus particularly on Lag 1 during the AB. Figure 1a depicts a classic AB experiment (Raymond *et al.* 1992; Chun and Potter 1995), in which a second target (T2) presented after a first target (T1) in an RSVP stream exhibits a deficit with a characteristic temporal profile (see Fig. 1c, green curve). That is, when there are between one and three distractors between the targets, T2 performance is impaired. Critically however, T2 performance at Lag 1 (no intervening distractors) is higher than that during the blink, i.e. performance is spared at Lag 1 (left-most point of green curve). In the AB experiment considered here, participants report the identity of the targets at the end of the RSVP stream. This identification report for T2 gives us the objective (Type-1) measure in this paper.

In this experiment, participants also reflected and reported on their visual experience of the T2 on a rating scale from 1 (‘Not seen’) to 6 (‘Maximal visibility’); see Fig. 1b, giving us our subjective (Type-2) measure. The resulting temporal profile of subjective visibility report (see Fig. 1c, blue curve) corresponds to the experiential blink (Pincham *et al.* 2016).

Comparing attentional and experiential blinks reveals a striking dissociation between what participants report consciously perceiving (quantified using subjective visibility) and what is encoded into working memory (quantified using identification report). This has been demonstrated experimentally by a strong interaction between subjective visibility and report accuracy carried by short lags of the AB curve (Jones *et al.* 2020); see Fig. 1c, blue (visibility) and green (accuracy) curves.

This dissociation suggests that working memory encoding is not impaired as the perceptual demands increase (i.e. the two targets are closer in time). Indeed, the data suggest that working memory of the second target is facilitated if it immediately follows the first target, i.e. there is Lag-1 sparing (left-most green data point in Fig. 1c). However, experience of the second target is increasingly impaired (Fig. 1c, blue curve) as the two targets are closer in time. This suggests a fundamental decoupling of encoding and experience that theories of meta-observers need to accommodate.

One objective of this paper is to link this phenomenon to what, taking inspiration from the metacognition literature (Fleming and Lau 2014), could be called ‘meta-experience’, i.e. how accurately one’s introspection and report upon a visual experience matches objective performance. Indeed, the meta-experience concept can be seen to respond to a long running critique of the relevance of classic metacognitive measures such as confidence: that they reflect the reliability of first-order judgements rather than phenomenology (Seth 2008). In contrast, the (Type-2) meta-measure associated with the meta-experience concept we consider here is subjective visibility, tying more directly to phenomenology.

This concept of meta-experience will enable us to provide evidence that the information (measured in bits) associated with an encoding into working memory (objective performance) ‘decouples’ from the experiential reflection upon that perceptual/encoding event (subjective performance) and that this decoupling is largest when there is the greatest perceptual overload (i.e. at Lag 1). That is, we will be able to show that at Lag 1 there is little difference in objective performance between trials when participants report high and low visibility, suggesting low meta-experience. Taking inspiration from the AB and our previous identification of an experiential blink (Pincham *et al.* 2016; Jones *et al.* 2020), we call this the ‘meta-experiential blink’.

A mechanistic interpretation of this finding is that, during such perceptually overloaded events, a meta-experiential self-observer is only able to observe single coherent items at a time, while multiple items can be encoding into working memory. This is the core of the ‘Simultaneous Encoding/Serial Experience’ (SESE) hypothesis.

We assess how our meta-experiential blink data could be modelled using self-observation theories. We do this by (i) providing model fits in the simultaneous type/serial token (STST) model of temporal attention and working memory encoding (Bowman and Wyble 2007; Bowman *et al.* 2008; Wyble *et al.* 2009), since it is computationally instantiated and a prominent model of the AB, as well as simulating electroencephalogram (EEG) data and (ii) adding a meta level to STST, giving us the ‘readout-enhanced Simultaneous Type/Serial Token (reSTST)’ model, we are able to model the meta-experiential blink and associated P3 EEG responses. Then, (iii) we present predictions (including counter-intuitive ones) that the model makes, which can be the focus of future empirical investigation.

## Background

### The experiential blink

The AB (Raymond *et al.* 1992; Chun and Potter 1995) is typically observed when two target stimuli (T1 and T2) are placed within an RSVP stream of stimuli (see Fig. 1a). Here, objective performance is quantified with end-of-stream report of the identity of the T1 and T2 letters; this is what we describe as the ‘report-accuracy’ measure. The green curve in Fig. 1c shows a typical AB: the probability of reporting the identity of T2 correctly, given correct report of T1 [i.e. *P*(T2|T1)] is reduced when there are between one and three distractors between the two targets. Thus, the processing of T2 is impaired while T1 is being processed.

The ‘experiential blink’ (Pincham *et al.* 2016) quantifies how the visibility of T2 (given correct report of T1) changes with lag; see the visibility scale in Fig. 1b and the blue curve in Fig. 1c, and also Sergent and Dehaene (2004). Thus, the attentional and experiential blinks involve the same stimulus presentations, but they concern different end of each stream reports.

Previously, we have highlighted the possibility of dissociation between working memory encoding and subjective experience of T2 (given correct report of T1) during the AB (Pincham *et al.* 2016; Jones *et al.* 2020). Specifically, as shown in Fig. 1c, at long lags (4 onwards) subjective report and report accuracy track each other with a fixed offset, but as lag reduces from 3 downwards, subjective report decreases, while report accuracy increases, with the largest difference at Lag 1.^1^ This kick up in report accuracy performance is the familiar Lag-1 sparing phenomenon (Hommel and Akyürek 2005; Bowman and Wyble 2007; Wyble *et al.* 2009).

Supported by state-trace analyses (Jones *et al.* 2020), we have taken this finding to imply that the coupling between report accuracy and subjective visibility is lost at early lags. This raises the possibility that a phenomenon that might be called ‘sight-blind recall’ exists. That is, it may be possible in some perceptually very demanding situations, for items to be recalled without having been consciously seen, which would be consistent with the now large literature on working memory without conscious experience (Soto *et al.* 2011; Soto and Silvanto 2014; Trübutschek *et al.* 2017, 2019). In a sense, the dissociation we have identified may suggest a mechanism by which unconscious working memory representations are formed. We discuss the support for and implications of the sight-blind recall concept in Pincham *et al.* (2016) and Jones *et al.* (2020); particularly see the discussion sections. However, more empirical work needs to be performed before the sight-blind recall interpretation can definitively be affirmed.

### Simultaneous encoding/serial experience

Our proposal is that the decoupling of measures during the blink is the result of the working memory encoding of stimuli occurring simultaneously but their experiences occurring in serial. This would result in working memory encoding proceeding simultaneously as the two targets approach one another in time, i.e. as lag decreases (c.f. Lag-1 sparing during the classic AB, left-most data point in green curve in Fig. 1c), while subjective report worsens (blue curve in Fig. 1c) because of the temporal proximity of the second target to the first.

This could work as follows. Suppose there is a ‘readout’ from some late stage of visual processing that indexes subjective experience, i.e. how visible a stimulus was, yielding an activation response that we call the ‘intermediate-trace’. We say that when the readout registers a response above some (visibility) threshold, a stimulus is being consciously experienced, and in all other cases it is not. We then, as per our hypothesis, assume a seriality of this readout: a second stimulus cannot begin to be consciously experienced until the previous stimulus falls back below threshold.

**Figure 2.**
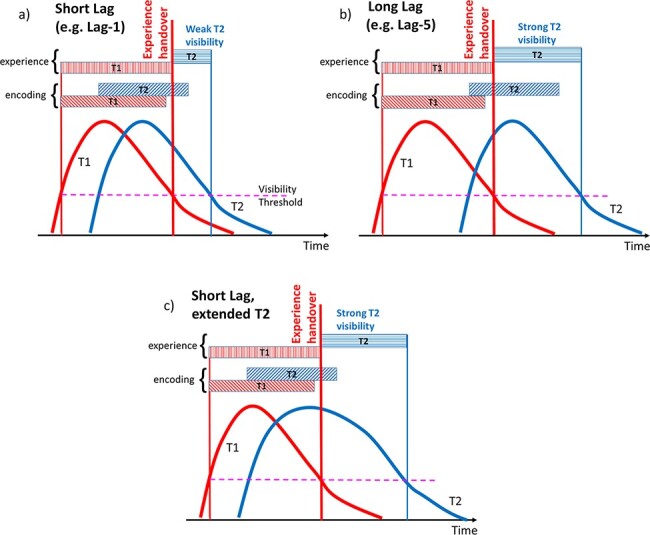
SESE theory. Horizontal bars above activation traces illustrate assumed time periods of encoding or experience for T1 and T2. Thick vertical red line indicates termination of T1 experience and onset of T2 experience, i.e. experience handover. (a) Short T1–T2 lag: although the amplitude and form of the response of both stimuli are the same, the duration of the experience of the second stimulus is greatly reduced because it cannot start being experienced until the first stimulus falls below the visibility threshold. By that time, T2 activation has decayed to the point that it only very briefly drives experience (short blue bar). In contrast, in (b) long T1–T2 lag, the amplitude and form of the response to both stimuli remain the same, but the time delay between first and second targets is increased, and consequently they are both experienced for similar durations. In (c) extended T2 trace, T2’s response is longer; consequently T1 and T2 are again both experienced for similar durations

When two targets are presented close together—say at Lag 1—there is typically a poor experience of the second target because, despite a potentially strong representation at the late stage of visual processing, most of its readout is subsumed by its temporal overlap to the first target. This is the situation depicted in Fig. 2a. Conversely, if the two targets are far apart—say at Lag 5—a second target with the same representation strength will be experienced much more strongly because its experience is no longer dominated by the temporal overlap to the first target. This is the situation depicted in Fig. 2b. Additionally, as shown in Fig. 2c, even in the case in which the two targets are closely presented, a sufficiently elongated activation trace of the second target allows the visibilities of both targets to be similar.

Under such a system, average visibility will increase as the temporal distance between the two targets increases, up to an upper limit at which the two targets are sufficiently far apart that the second is not materially affected by its temporal proximity to the first. This is the kind of situation we see in Fig. 1b.

### Meta-measures

Importantly, previous experiential blink work has been based upon relating ‘mean’ report accuracy to ‘mean’ subjective visibility and considering how that relationship changes with lag (Pincham *et al.* 2016; Jones *et al.* 2020). What has not been done is to decompose the accuracy and visibility measures to understand the coupling between them. If the SESE theory holds, then the probability of responding correctly for low-visibility trials should increase as lag decreases (i.e. we move from Panel b to a in Fig. 2), and certainly that probability should increase faster for low-visibility (Fig. 2a) than high-visibility (Fig. 2c) trials as lag decreases.

In response, we propose our ‘meta-experience’ measure, whereby, taking inspiration from measures of metacognition (Fleming and Lau 2014), we have both Type-1 and Type-2 measures. However, for meta-experience, the Type-2 measure is subjective visibility, rather than confidence, taken as a measure of the vividness with which a stimulus is consciously perceived (cf. Lau and Passingham 2006; Rounis *et al.* 2010, for related approaches).

Metacognition approaches are typically framed within signal detection theory (Maniscalco and Lau 2012; Fleming and Lau 2014), requiring the Type-1 task to be either a detection task, or decomposable into a binary choice. This is not the case in our AB task, which entails target identification involving 21 different stimuli and outcomes.

We therefore develop two new approaches to quantifying metacognitive sensitivity, the first based upon the ‘information’ carried by a pattern of responding and the second based upon the ‘correctness’ of responses. (Although similar, these two approaches are not actually the same.) As it is new in this domain, we elaborate on ‘Discrete Mutual Information’ [hereafter mutual information (MI)].

### Mutual information

MI calculates sensitivity (i.e. discriminability), without being concerned with the criteria that participants use to distinguish stimuli or, even strictly, how well responses correctly correspond to stimuli. Rather, MI assesses how ‘systematically’ participants’ responses discriminate between the stimuli presented, i.e. how much information responses carry about stimuli. In other words, if one knows a response, how effectively can one know the eliciting stimulus? It measures this in units of bits of information. More precisely, MI quantifies the amount of information that one (discrete) probability distribution $X$ tells us about another (discrete) probability distribution $Y$. That is, with joint distribution $\left( {X,{\ }Y} \right)$, the MI of $X$ and $Y$ is defined as:

$$I\left( {X;{\ }Y} \right){\ } = {\ }H\left( X \right){\ } + {\ }H\left( Y \right){\ } - {\ }H\left( {X,{\ }Y} \right)$$

where *H* is the entropy. Thus, MI is high when the two marginal distributions have high entropy (i.e. there is a good deal of uncertainty to resolve), but the joint entropy is small (i.e. there is a systematic relationship between the values of the two marginal distributions). Further details of MI are given in Supplementary Material 1.

We apply MI to stimulus/response correspondence; i.e. in the above definition, *X* would be presented T2 stimuli and *Y* would be T2 identification responses, in both cases letters of the alphabet. In this way, MI serves as a measure of objective (i.e. Type 1) performance, i.e. the amount of information that end-of-stream identification report carries about the presented T2.

### Meta-experience

We can divide visibility report into high/low-visibility bins: giving us a 2-point scale for visibility: High and Low (Note: there are other good reasons for wanting to collapse visibility bins, see Supplementary Material 2). This gives us our Type-2 measure: whether the participant reported seeing the presented T2 well (high visibility) or poorly (low visibility).

We define **‘**Meta-experience’ as the relationship between these two measures. Furthermore, since we have collapsed visibility into two bins, we can formulate meta-experience as the difference of MI between trials that yield a high-visibility response and trials that yield a low-visibility response.

Thus, as the information extracted for high visibility increases relative to the information extracted for low visibility, the Type-2 measure (subjective visibility) is increasingly strongly coupled to the Type-1 (objective) measure (here MI). In other words, when the participant reports high visibility, their brain has objectively extracted more information about the presented stimulus than when they report low visibility. In contrast, if there is little difference between the MI observed for high-visibility versus low-visibility trials, the Type-2 measure is decoupled from the Type-1 measure, and subjective visibility report is telling us very little about the objective information extracted by the brain.

We can also replace MI with report accuracy in our formulation of meta-experience, giving us a measure which we call meta-experience^A^. However, the resulting meta-experience scales are different, since high MI does not necessarily imply correct responding, an issue we discuss later.

## Materials and methods

### Readout-enhanced STST model

The SESE hypothesis has been realized in the ‘readout-enhanced STST’ model (Jones *et al.* 2020). This takes the STST model, as defined in Bowman and Wyble (2007), and adds a subjective visibility readout mechanism and an associated revised means to generate virtual/synthetic event-related potentials (ERPs). However, importantly, the functionality and parameter settings of the previously published model, which we call the ‘core’ model, are not changed. We do, however, select a slightly different stimulus range to our previous virtual ERP work, e.g. Craston *et al.* (2009). Specifically, we sample (uniformly) a range of stimulus strengths with greater variability (−0.078 to +0.078 becomes −0.1625 to +0.1625), at a slightly higher average distractor and T2 strength (0.520 becomes 0.570). Additionally, T1 and T2 have different strengths in our simulation. This is because the Pincham *et al.* (2016) experiments used a colour-marked T1, which will create a strong onset. Accordingly, the T1 is modelled with an average strength of 0.670.

This approach of using new stimulus strengths is consistent with previous STST simulations, where input strength ranges have been allowed to vary, reflecting the fact that different experiments being modelled might have quite different stimulus types and sensitivities. (See Jones (2020), especially Appendix C for further discussion of changes made, and Supplementary Material 3 for further details of the visibility calculation we add here).

This ensures that the results presented here are consistent with previously reported STST fits to data, of which there are now many (Bowman and Wyble 2007; Bowman *et al.* 2008; Chennu *et al.* 2009; Craston *et al.* 2009; Wyble *et al.* 2009, 2011). Additionally, the capacity to exhibit further effects without refitting parameters suggests that those originally identified parameter settings were not ‘over-fit’ in a particularly obscure and unrepresentative region of parameter space (Roberts and Pashler 2000), i.e. the parameter fits ‘generalise’ to a new set of phenomena.

**Figure 3.**
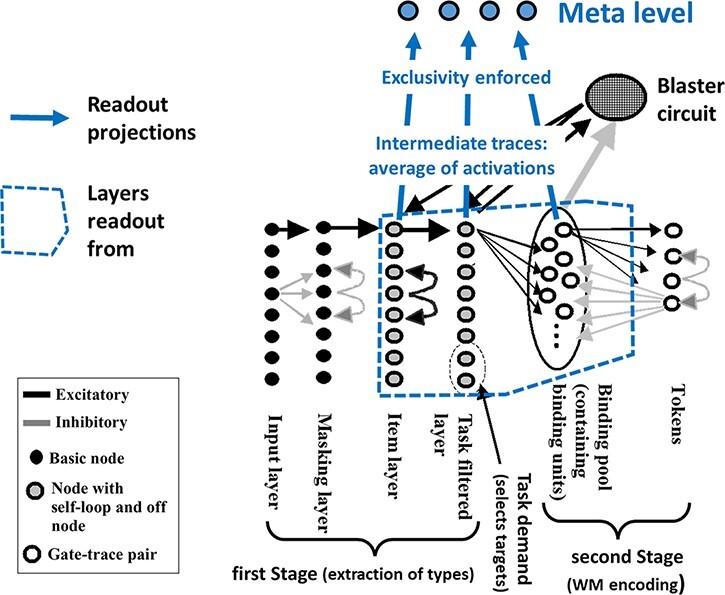
Readout enhancement of the STST model (Bowman and Wyble 2007). For full details of the STST model, please refer to Bowman and Wyble (2007). For the purposes of this paper, the first stage can be viewed as the brain’s visual processing pathway (specifically, the ventral stream). The second stage implements working memory encoding, by associating a Stage-1 representation with an episodic tag (called a token). This association is constructed through a binding pool, which could be viewed as a potential implementation of the global workspace (Dehaene *et al.* 2003). The readout additions to the basic STST model are presented in blue. Excitatory units in the region demarcated by dashed blue project to a meta-level layer. This meta level receives intermediate traces, which are linear sums of activation from readout layers of the STST model, subject to enforcement of exclusivity to ensure serial experience

This readout-enhancement mechanism is fully described in Jones *et al.* (2020) and depicted in Fig. 3. It is summarized in the following points.

1. The source of the subjective visibility measure is the average of post-synaptic potentials across late layers of the core STST model; see the blue dashed annotations in Fig. 3. We call the resulting activation traces ‘intermediate-traces’, since they sit between sensory pathways and readout.
2. When the intermediate trace for a stimulus is above a given amplitude (the threshold of subjectivity), it could be being ‘subjectively experienced’ and when it is below, it is certainly not. Additionally, this experience is serial. If the individual intermediate traces for two stimuli are both above the threshold, then the second in time cannot be experienced until the intermediate trace for the first has fallen below threshold. In this sense, exclusivity is enforced; see readout additions and meta level in Fig. 3 [Note that this is quite like the ‘mutual exclusion’ enforced in concurrent and distributed computer systems (Dijkstra 2001)].
3. The strength of an item’s subjective experience is determined by the maximum amplitude of its (above threshold) intermediate trace, subject to no other stimulus already being above the subjective visibility threshold. Thus, this maximum amplitude determines its subjective visibility, i.e. its visual vividness. This represents a particularly simple quantity to base visibility upon, which should be preferred under Occam’s Razor.
4. To reflect neurophysiological noise, we add two sources of Gaussian distributed noise, one additive and the other multiplicative. The second of these is the most important, reflecting the presence of multiplicative noise in neuroimaging data; see, e.g. the Fano factor (Eden and Kramer 2010). This source of variability also avoids the somewhat counter-intuitive property of additive noise that it can (in admittedly rare cases) turn a zero-amplitude activation trace into a high-amplitude, high-visibility percept, i.e. induce an illusory percept. Since zero multiplied by anything is zero, such illusory percepts cannot be generated by a multiplicative source of noise. Note that the addition of noise to the readout process, which enables us to fit the meta-experience data better, is new to this paper. Accordingly, the behavioural and ERP fits presented here, for what we call the ‘noisy readout-enhanced STST model’, are somewhat different to those in Jones *et al.* (2020).

Further details of the readout mechanism and particular parameter settings are presented in Supplementary Material 3.

In addition to being compared to behaviour, the model presented here is compared to human ERPs, through what we call virtual ERPs. The virtual ERPs generated in Craston *et al.* (2009) were calculated by summing the post-synaptic activation of all relevant excitatory units together. In the model we present here, when a first target’s activation trace crosses the threshold of subjectivity, it starts contributing to the P3; however, the activation traces of other targets do not contribute to the P3. This reflects our position that the P3 corresponds to conscious experience rather than working memory (WM) encoding (Pincham *et al.* 2016) and is the same as the approach employed in Jones *et al.* (2020), where a full description is given in Supplementary Material Section D of that paper.

Additionally, we smooth the virtual ERPs generated from the model with a Gaussian kernel. This is justified by the scalp acting as a low-pass filter between brain sources and electrodes in human scalp EEG.

Since the reSTST model does not have a concept of target identity, creating a stimulus–response matrix, in order to calculate MI, as described in previous sections is not possible. Consequently, we are only able to compare the model to accuracy for high and low visibility rather than to MI.

### Experimental paradigm

We analyse and model two data sets from experiments first published in Pincham *et al.* (2016), in which both working memory encoding and subjective experience are measured during the AB. The first experiment examined behavioural responses and assessed a larger number of lags (Lags 1, 2, 3, 4, 6 or 8) in order to sample the full AB curve. The second collected both EEG and behavioural data, with only Lags 1 and 3 sampled for 80% of trials in order to enhance the EEG signal-to-noise ratio.

Targets were uppercase letters and distractors were single digits; the first target (T1) was always presented in red and the second (T2) in white (see Fig. 1a). Stimulus Onset Asynchrony (SOA) was 90 ms. At the end of each RSVP stream, participants rated the subjective visibility of T2 using a 6-point self-report scale (see Fig. 1b). Participants then reported the identity of the T1 and T2 (therefore, d and a would be the correct responses for the stream in Fig. 1a). Participants were required to guess if they were unsure of the target identities. Full details can be found in Pincham *et al.* (2016).

### Statistical considerations

Since entropy is a biased measure with small samples, we use the NSB (Nemenman, Shafee, Bialek) estimator (Nemenman *et al.* 2002), which is justified in Supplementary Material 2. Despite this, some bias may still remain, particularly at the sample sizes we are using. However, from the investigations in Jones (2020), with *s* the number of bins (21 for us: the number of stimuli and of responses) and a sample size of *n*, in the worst case, the bias is *O*(*s*/*n*)* = O*(21/*n*) (Paninski 2003). Thus, we can estimate the error for each of our samples and add these as covariates of no interest to our statistical model. Additionally, for a sample size of 0, MI is not defined. Accordingly, we employ mixed effects models, which are robust to missing data (Krueger and Tian 2004).

### Inferential statistics

In order to determine the effects of our factors on MI, we fitted linear regression mixed models [using the R lme4 package (Bates *et al.* 2007)]. The dependent measure in all of these models was MI, calculated as $\text{MI}\left( {X;{\ }Y} \right){\ } = {\ }{H_\text{{NSB}}}\left( X \right){\ } + {\ }{H_\text{{NSB}}}\left( Y \right){\ } - {\ }{H_\text{{NSB}}}\left( {X,Y} \right)$, with ${H_{NSB}}$ the NSB estimator of entropy. Independent measures were lag (Lag), visibility bin (Vis), lag/visibility interaction (Lag × Vis), count (the reciprocal of the sample size, our estimator of entropy bias—a covariate of no interest) and subject (Subject). Lag and visibility were both categorical variables and were dummy coded with respect to Lag 1 and low visibility, respectively. Lag, visibility bin, lag/visibility interaction and count were fixed effects; Subject was a random effect on the intercept. We consider five models, which we denote using the notation from the lme4 package:

$$\text{Null:}{\ }\text{MI}{\ } = {\ }1{\ } + {\ }\text{Count}{\ } + {\ }\left( {1|\text{Subject}} \right)$$

$$\text{Lag:}{\ }\text{MI}{\ } = {\ }1{\ } + {\ }\text{Lag}{\ } + {\ }\text{Count}{\ } + {\ }\left( {1|\text{Subject}} \right)$$

$$\text{Vis:}{\ }\text{MI}{\ } = {\ }1{\ } + {\ }\text{Vis}{\ } + {\ }\text{Count}{\ } + {\ }\left( {1|\text{Subject}} \right)$$

$$\text{allMain:}{\ }\text{MI}{\ } = {\ }1{\ } + {\ }\text{Lag}{\ } + {\ }\text{Vis}{\ } + {\ }\text{Count}{\ } + {\ }\left( {1|\text{Subject}} \right)$$

$$\text{Full:}{\ }\text{MI}{\ } = {\ }1{\ } + {\ }\text{Lag}{\ } + {\ }\text{Vis}{\ } + {\ }\text{Lag}{\ } \times {\ }\text{Vis}{\ } + {\ }\text{Count}{\ } + {\ }\left( {1|\text{Subject}} \right)$$

Models were compared using a chi-square test. For the main effect of lag, which determines if an AB is present, we compared the Lag model with the Null model. For the main effect of visibility, which determines if an overall meta-experiential effect is present, we compared the Vis model with the Null model. For the Lag-by-Vis interaction, which assesses whether meta-experience changes with lag, we compared the Full model with the allMain model and the Null model separately.

In order to provide a more highly powered test of the presence of an AB, we also compared Lags 2, 3 and 4 collapsed (during the blink) to Lags 6 and 8 collapsed (recovery from the blink). Since we have a very strong precedent for the direction of this binary effect—inside the blink lower than outside—we also ran this one-tailed. Since the lme4 package is not set up to perform such a one-tailed test, we ran this as a simple one-tailed two-sample paired *t*-test.

## Results

### Experiential blink (new fits)

#### Behaviour

Figure 1 compares report accuracies and subjective visibilities predicted by the (noisy) reSTST model (reSTST), see Panel d, to those from the human data (Panel c). Although there are differences between human and model findings, the qualitative pattern of results is similar. The STST model demonstrates a somewhat more marked downturn in subjective report at earlier lags than the human data (compare blue curves). This may suggest that the decay rate of T2s, which have to wait until the T1 has completed, is relatively fast in the model [however, see Avilés *et al.* (2020), for evidence of fast decay in humans].

#### Event-related potentials

**Figure 4.**
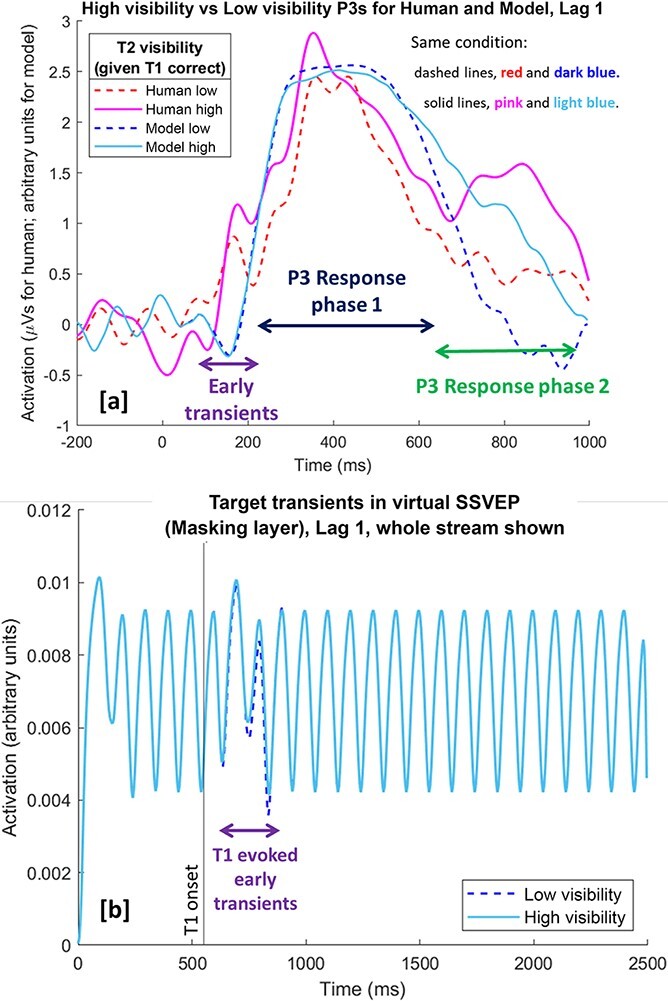
A comparison of human ERPs (pink and red) and virtual ERPs (blues) generated from the noisy readout model for high- and low-visibility T2s, at Lag 1. Note that in all these ERPs, the T1 was correctly reported, and in a it is reflected in Response Phase 1. [a] Comparison of P3s at Lag 1: The virtual ERPs presented here are new fits and somewhat different to those presented in Jones *et al.* (2020), since we are using the noisy reSTST model. Zero on *x*-axis is the point of T1 onset. Human ERPs are recorded from the Pz electrode. [b] SSVEP generated by model (activation from Masking layer, which is the model’s analogue of Iconic memory). Since we are depicting the whole period of the stream, the time axis here is different to that in a. The extra strength of the T1 stimulus, which is simulating a colour-marked T1 in Pincham *et al.* (2016), evokes a transient in the SSVEP, which could be related to the early transients indicated in the human data in Panel a. It is only during this transient that the low-visibility condition does not perfectly follow the high-visibility condition and as a result is obscured by it

We compare the human (Lag-1) ERPs (recorded at Pz) to the model (Lag-1) ERPs in Fig. 4. There are two seeming differences between the two: (i) early transients (100 ms to 250 ms approx.) and (ii) late dynamics (from 650 ms approx.). We discuss these in turn shortly.

In general, however, there is again a good ‘qualitative’ fit between the (noisy) reSTST data and the human data, which as we argue elsewhere in this article is the appropriate criterion for comparing model and human, given that core model parameters fixed in Bowman and Wyble (2007) have not been changed. Most importantly, our simulation results provide a proof of principle that the explanation presented in Fig. 2 (regarding why report accuracy and subjective visibility diverge at Lag 1) is tenable. This explanation rests on the proposal that encoding into working memory can proceed in parallel, but subjective experience cannot.

##### Early transients

There are differences between human and model ERPs before 250 ms; see early transients marked in Fig. 4a. These are likely to be due to the stronger onset arising from the colour-marked T1 in the human data. As previously discussed, this stronger onset is modelled by giving the T1 higher strength than the T2 or distractors. This creates a distinct (phasic) transient in the steady-state visually evoked potential (SSVEP) generated by STST; see Fig. 4b. We believe that it is these transients, probably due to volume conduction, bleeding into the Pz electrode that generate the early transients observed in the human ERPs.

##### Late dynamics

The most important feature in the human ERPs is the extended P3 when a T2 is seen vividly (High Vis condition), c.f. pink filled line substantially higher than red dashed line in Response Phase 2. Thus, a clear conscious percept of T2 coincides with a ‘longer’ P3, rather than a dramatically higher amplitude in Response Phase 1. This is the serial experience property of SESE, i.e. the T2 has to wait until the T1 has completed being experienced (in Response Phase 1), before it can be experienced. This implies that the P3 is a correlate of conscious perception rather than working memory encoding; c.f. Pincham *et al.* (2016) for a discussion of the debate on this issue.

Additionally, the important property that a clear conscious percept of T2 (i.e. the high-visibility condition) coincides with a longer P3 is qualitatively present in the virtual ERP in Fig. 4a, filled light blue higher than dashed dark blue in Response Phase 2. There are however some differences between the late dynamics of the noisy reSTST model and the human data, with the noisy reSTST ERPs showing differences to the human data from approximately 650 ms onward. Specifically, in this second phase, the model tends to generate an extended P3 in the T2 high-visibility condition (filled light blue line) rather than the (at least partially) distinct P3 that looks to be present in the human data (filled pink line). This may suggest a less efficient transition from experiencing T1 to experiencing T2 than is presently implemented in the model, thereby generating a temporal gap between the two. However, we await further ERP data before concluding that the model needs to be adjusted in this respect.

### Meta-experience

#### Human behaviour

We measure meta-experience as the difference between MI for high and low visibility, which we plot by lag. We also plot a similar difference in accuracy rates by lag for high and low visibility (which we refer to as meta-experience^A^).

**Figure 5.**
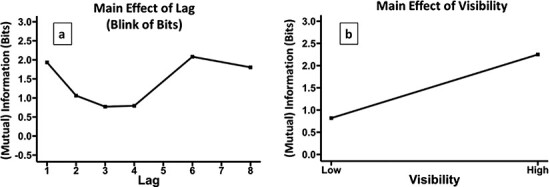
Main effects for T2, given T1 is correctly reported. (a) The effect of lag, reflecting how the information extracted (MI) from the RSVP stream about the T2 changes with lag. This can be interpreted as how systematically participants respond at each lag. This is the blink of bits, i.e. the number of bits of information extracted at different lags. Note that the dip in Lag 8 is a common feature of RSVP experiments that sample longer lags more sparsely than shorter lags, i.e. participants get used to seeing early lags and thus allocate their temporal attention less to late lags. (b) The effect of visibility, i.e. how MI changes with visibility. MI depicted in (a) and (b) is calculated from a single stimulus–response matrix that is constructed from responses across all subjects, i.e. with subject collapsed out. This generates a denser stimulus–response matrix from which MI can be calculated with more accuracy. This is done just for visual clarity; all statistics were run with subject as the unit of inference

Our Lag model explained 15% of the variance in MI and approached being significantly different from the null model, $x$^2^(5) = 10.851, *P* = 0.05441; see Fig. 5a. For our higher powered test of the presence of a blink of information, which compared inside and outside the blink, the classic paired *t*-test we ran was significant [*t*(17) = −2.52, *P* = 0.011]. The Visibility model explained 49% of the variance and was significantly better than the null model $x$^2^(5) = 105.59, *P* < 0.001; see Fig. 5b.

**Figure 6.**
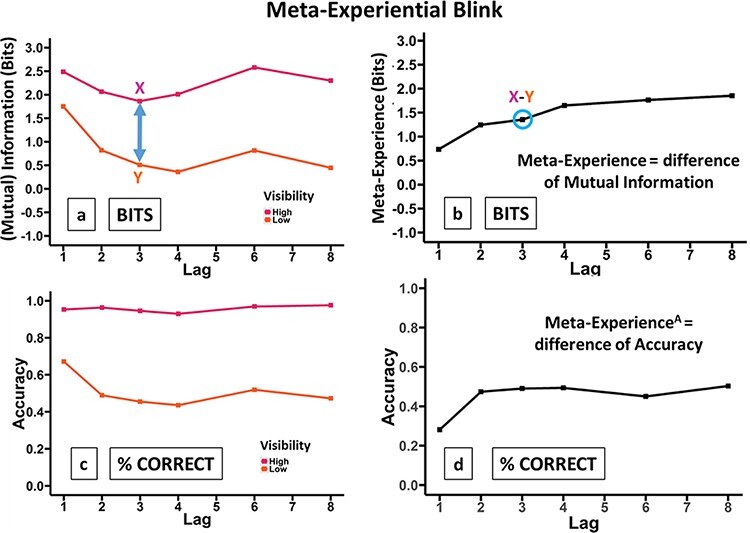
The meta-experiential blink, depicting T2, given T1 is correctly reported. The effect of lag on meta-experience is shown. (a) Decomposition into high- and low-visibility blinks, where the difference in MI between these at any lag, illustrated for Lag 3, is meta-experience. (b) Summarization of Panel (a): meta-experience is plotted directly, with meta-experience strikingly low at Lag 1, relative to other lags. MI depicted here is calculated from a single stimulus–response matrix that is constructed from responses across all subjects, i.e. with subject collapsed out. This generates a denser stimulus–response matrix from which MI can be calculated with more accuracy. This is done just for visual clarity; in particular, all statistics were run with subject as the unit of inference. (c and d) The corresponding plots to a and b but for (percent correct) accuracy. This gives us the meta-experiential blink for accuracy

The Full model explained 63% of the MI variance and was better than both the null model $x$^2^(5) = 167.62, *P* < 0.001, and the allMain model $x$^2^(5) = 36.128, *P* = 8.9 × 10^−7^. The latter of these implies the presence of the Lag-by-Vis interaction, i.e. that the difference in MI between high- and low-visibility changes with lag. This is the meta-experiential blink, i.e. the link between visibility levels and information changes with lag. We depict this in two corresponding forms in Fig. 6. Importantly, meta-experience at Lag 1 is strikingly low relative to other lags.

**Figure 7.**
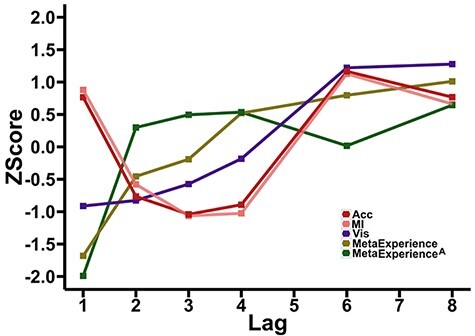
Serial-position curves for accuracy, MI, visibility, meta-experience and meta-experience^A^ for T2, given T1 is correctly reported. All measures are *z*-scored relative to the distribution set-up by the six lags to enable a direct comparison of these different measures. Accuracy and MI track each other relatively closely, mapping out classic AB patterns, with prominent Lag-1 sparing. As reported in Pincham *et al.* (2016) and Jones *et al.* (2020), subjective visibility exhibits a monotonically decreasing pattern as lag decreases, with no Lag-1 sparing (i.e. Lag-1 impairing). The meta-experience curves are also essentially monotonically decreasing as lag decreases and exhibit strong Lag-1 impairing

Figure 6c and d shows that calculating meta-experience from accuracy (our meta-experience^A^ measure), rather than information (Fig. 6a and b), leads to a similar overall trend. In particular, meta-experience at Lag 1 is again dramatically smaller than that at Lag 2. There are, however, some interesting differences in the relationship with lag, which we return to in Discussion. We also compare the serial-position curves for all the relevant measures in Fig. 7.

As can be seen in Fig. 7, the meta-experiential blink (green curves) is strongly concave, when compared with the (convex) experiential blink (purple curve showing visibility). That is, as lags get smaller from 4 to 3 to 2 to 1, for the experiential blink, the amount performance reduces themselves reduce, but for the meta-experiential blink, the amount performance reduces get larger (especially from Lag 2 to Lag 1). This suggests that relatively speaking, Lag 1 is even more dramatically impaired for meta-experience than for experience. This could be summarized as saying that the closest proximity of T2 to T1 leaves, (i) encoding into working memory unimpaired (indeed facilitated relative to blink recovery; see No Blank condition in Fig. 4 of Chun and Potter 1995); (ii) conscious experience impaired and (iii) the capacity to reflect on what enters working memory on the basis of conscious experience even more impaired.

#### Model

**Figure 8.**
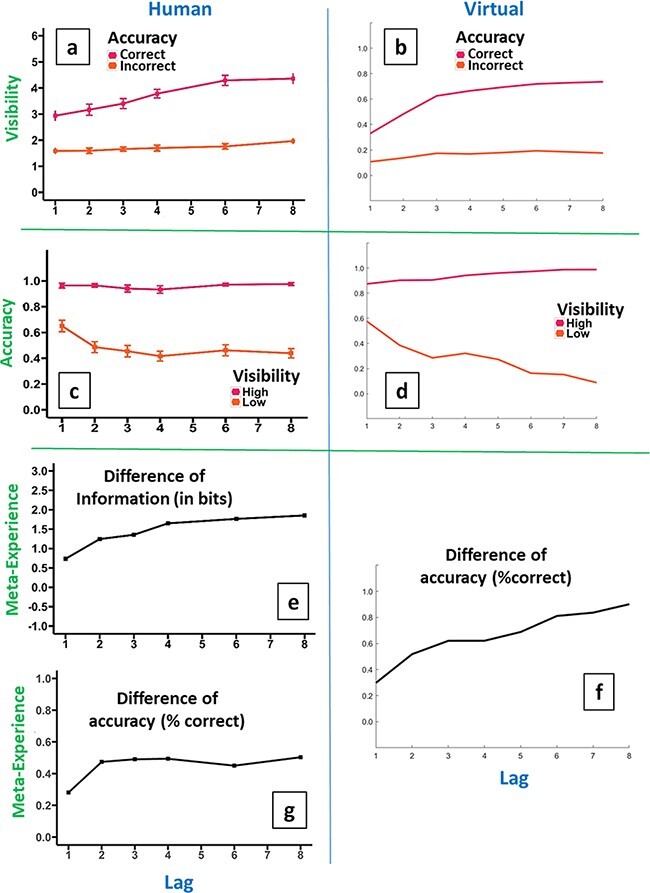
Comparison of human and (noisy) reSTST model: left side shows human data, right side shows model fits. First row: mean visibility serial-position curves for correct and for incorrect response trials in humans (a) and model (b). Both humans and model exhibit a largely constant visibility for incorrect responses but a decreasing visibility for correct responses as lag decreases (from 5 down to 1). Second row: mean accuracy serial-position curves for high- and low-visibility trials in humans (c) and model (d). Both exhibit the largest change in the low-visibility trials, with accuracy climbing substantially as lag decreases at small serial positions (3 to 2 to 1) for both and somewhat for later lags in the model. Bottom three panels: meta-experience serial-position curves in humans (e and g) and model (f, difference of accuracies). All essentially exhibit decreases in meta-experience as lag reduces at small serial positions (3 to 2 to 1), while the model (and to some extent information, Panel e) exhibits decreases at longer lags as well; Panel f

We compare the behavioural predictions that the (noisy) reSTST model makes concerning meta-experience to those from human data, see Fig. 8, with human data on the left and model output on the right. Importantly, we have not refit the (core) STST model to these data, rather, as we have done in all our work with the model, we have maintained the parameter settings associated with its first publication (Bowman and Wyble 2007). As a result, it is only reasonable to expect the model to be qualitatively accurate.

In this qualitative sense, the match of model to human data is good. We can identify the following patterns in the data in Fig. 8,

1. Top row (Panels a and b): both humans and model exhibit a largely constant visibility for incorrect responses but a decreasing visibility for correct responses as lag decreases (Lags 5 to 1).
2. Middle row (Panels c and d): for both humans and model, the largest change is in the low-visibility trials, with accuracy climbing substantially as lag decreases at small serial positions (3 to 2 to 1).
3. Bottom row (e, g and f): both humans and model exhibit decreases in meta-experience as lag reduces at small serial positions (3 to 2 to 1).

## Discussion

One way to attempt to understand a system is to explore how its behaviour degrades when it is overloaded. This approach can be applied to understanding conscious perception by e.g. presenting stimuli in rapid succession in the ‘same’ perceptual event/moment, e.g. Lag-1 in the AB (Hommel and Akyürek 2005; Wyble *et al.* 2009), temporal conjunction errors (Botella *et al.* 2001; Chennu *et al.* 2011) and the perceptual moment (Di Lollo and Wilson 1978). This paper has focussed on the Lag-1 case. This is because of our previous work, which has noted that the capacity to extract and encode identities of targets seems to increase, while the visual percept of those targets experienced seems to decrease, as they are presented closer together in time. In order to formally assess the coupling between memory encoding and perceptual vividness, we have introduced a new measure that we call meta-experience.

### A meta-experiential blink

Meta-experience quantifies the extent to which the (subjective) vividness of a percept reflects the (objective) information extracted by the brain. In a similar way to Rounis *et al.* (2010), meta-experience is a metacognitive measure focussed specifically on perceptual experience rather than decision confidence. Importantly, decision confidence could be affected by factors unrelated to perceptual experience, whereas meta-experience focusses on the dissociation between objective (i.e. information) and subjective experience, where the latter specifically refers to the perceptual experience of a stimulus.

We find that meta-experience decreases monotonically with decreasing lag all the way down to Lag 1, at which point it shows a particularly sharp downturn, generating a concave pattern (see Fig. 7, green curves). This suggests a general meta-experiential failure: although (objective) report accuracy on the second target increases at Lag 1 (Fig. 7, red and pink curves), there is a generalized and increasing failure to introspectively assess this performance as the two targets come closer together.

The sight-blind recall hypothesis gives a specific reason for this loss of introspective ability as lag decreases towards 1, viz that it results from participants systematically reporting poor (Type-2) visibility despite high (Type-1) identification performance. This would manifest as MI for high visibility increasing at a lower rate than MI for low visibility as lag decreases towards 1. This is what we observe in Fig. 6a. We would expect a similar pattern for report accuracy, with report accuracy for high visibility increasing at a slower rate than report accuracy for low visibility as lag decreases towards 1. Figure 6c shows this pattern, in fact, with report accuracy not increasing at all for high visibility, perhaps due to ceiling. Effectively, another phrasing of this is that we would expect the average visibility for correct trials to decrease as lag decreases, while the average visibility for incorrect trials would decrease at a slower rate or not at all. This is what we see in Fig. 8a.

### Blindsight

The dissociation between the (objective) AB and the (subjective) experiential blink that we observe is relevant to the blindsight literature, especially the search for blindsight in healthy observers, which has proved notoriously difficult to obtain (Peters and Lau 2015). Importantly, the objective performance we are focussed on here is free recall from working memory rather than forced choice performance, hence the term sight-blind ‘recall’. One might expect free recall to be especially closely tied to conscious processing. In addition, while our measure of visibility is subjective report and therefore affected by where subjects place their criterion for deciding between the different visibility levels, the key result here is a dissociation between performance and visibility as a function of lag. This would suggest that the decoupling would need to be explained by a shift in a (strategic) response bias applied only to the early subset of lags during a block; we discuss this possibility in the next subsection. The result however is also consistent with both a decrease in overall visibility, together with a decrease in meta-experience (the within-condition coupling between visibility ratings and performance) at earlier lags.

As previously indicated, our findings have some similarity to those presented in Rounis *et al.* (2010). Rounis *et al*. considered the coupling between visibility and accuracy to arrive at a similar quantification to our meta-experience measure. They demonstrated that theta-burst transcranial magnetic stimulation (TMS) to the dorsolateral prefrontal cortex impairs meta-experience in a fashion similar to blindsight. That is, TMS specifically reduces visibility for correct responses while not impacting incorrect responses. This is similar to our findings in Fig. 8a, where reducing lag (moving from right to left on *x*-axis) only has a very small impact on visibility report for incorrect responses but progressively and strongly reduces visibility for correct responses. This observation bolsters our claim that we have observed a phenomenon we call sight-blind recall.

Rounis *et al*. also argue that this pattern stands against a response-bias explanation of their findings since that would also impact the visibility judgement of incorrect responses. The analogous observation can be made for our findings. In particular, a tendency to be more conservative in visibility ratings at short lags, i.e. giving lower ratings, might be expected to be observed similarly strongly for incorrect responses. This said, more empirical work is required to definitively justify the sight-blind recall claim.

### Response bias: odd perception not poor perception

A potential criticism of the sight-blind-recall interpretation of our findings that certainly cannot be completely ruled out is that participants may be reporting low subjective visibility of the second target at short lags not because they perceived it poorly but because their percept was odd or unusual in some way. In this case, the peculiarity of the experience may cause participants to simply report low visibility. This would be the analogue for sight-blind recall of a central debate in blindsight research: whether patients’ high objective performance in the absence of reported awareness when stimuli are presented in their lesioned visual field could just amount to weak residual perception and a conservative response threshold on detection (i.e. on awareness report) (Michel and Lau 2021; Phillips 2021).

In our context, this would correspond to reduced subjective visibility ratings (i.e. more conservative responding) because the T2 percept is ‘odd’ at Lag 1. We extensively considered this potential confound and argue that it is unlikely to explain our findings in Pincham *et al.* (2016) and Jones *et al.* (2020). However, we re-iterate and elaborate on some of these points here, in the light of the meta-experience measure that we have brought to bear in this article.

The obvious analogue for sight-blind recall of the standard response-bias argument in blindsight is the possibility of experiencing integrated percepts at Lag 1. In particular, many have argued that Lag 1 is a special case, in which T1 and T2 are sometimes processed/perceived together, even as a single integrated percept (Hommel and Akyürek 2005; Bowman and Wyble 2007; Wyble *et al.* 2009; Akyürek *et al.* 2012). As just discussed, this raises the possibility that the reduction in subjective visibility ratings at Lag 1 is not specifically a reduction in conscious experience but more confusion about the conscious experience, leading, if you like, to a ‘loss of confidence’ in their experience.

Additionally, in and of themselves, our meta-experience findings do not alleviate this concern. For example, in Fig. 8a, visibility ratings reduce linearly from Lag 6 to Lag 1 for correct responses. This could arise from what might be considered a bias towards conservative responding to the subjective visibility question, i.e. lower visibility responses when actually a discriminable (in terms of enabling accurate objective responding) percept was experienced. [Although this particular pattern was not observed in a related study by Recht *et al.* (2019) (see ‘Experience at Lag 1’ subsection of this Discussion), on correct trials, they saw increased confidence at Lag 1, see their Fig. 3a.]

However, there are a number of reasons to believe that this confusion due to integration explanation cannot account for our findings. First, colour marking was incorporated in the experiment to ensure that T1 was coloured distinctly from the rest of the stream and particularly from T2. One reason for doing this was to mitigate against the possibility of obtaining integrated percepts at Lag 1, and there is evidence that this worked. Specifically, in letters-in-digits tasks, of which the experiment here is an example, the cardinal indicator of integrated percepts is order errors. That is, if T1 and T2 are encoded as a single undifferentiated whole, information about the order in which they occurred would be lost, and in fact, conjunction information between the T1, the T2 and which of them was colour-marked would be lost. Importantly however, order errors were very rare in our experiments: they were around 10% compared to 30% in classic, non-colour-marked letters-in-digits tasks (e.g. Chun and Potter 1995).

This stands against the suggestion that T1–T2 integrations are prevalent at Lag 1 and underlie the drop in relative subjective visibility at early lags in our experiment. Second, it is important to note that the reduction in relative subjective visibility can also be observed at Lag 2 and perhaps also the beginning of the effect at Lag 3, see Fig. 1c. The integration argument is however classically ascribed specifically to Lag 1 and not later lags, in which there are intervening distractors.

Third, we have endeavoured to perform an analysis uncontaminated by integration trials. This is presented as the fourth analysis in Supplementary Material C of Pincham *et al.* (2016). Under this analysis, the interaction effect between measure type (report accuracy and subjective visibility) and lag remained significant. This is strong evidence that our findings are not explainable by integrated percepts at Lag 1.

Fourth, the ERP findings do not obviously fit with the integrated percept explanation. In this context, the critical condition is T2 correct/low visibility, since this is the disparity case (T2 was correctly reported but was poorly perceived consciously). The prevalence of these trials at Lag 1 is what causes the relative reduction in subjective visibility at that serial position. If perceptual integration were characteristic of this phenomenon at Lag 1, we would expect to see an ERP reflecting this integration. The obvious candidate pattern for this situation is a relatively short but relatively high-amplitude P3, and indeed, this is the pattern normally observed for T1 and T2 correct versus T1 and not T2 correct at Lag 1 (see, e.g. Craston *et al.* 2009, Fig. 7). The slightly higher amplitude arises because T1 and T2 are being encoded at the same time, and the relatively short positive deflection (not much longer than for T1 and not T2) arises from the simultaneity of encoding, i.e. T2 does not have to wait for T1 to be encoded, which would generate a very long positive deflection. Importantly, the ERP we observe for T2 correct/low visibility, see Fig. 6, Panel A of Pincham *et al.* (2016), does not follow this classic integration pattern. In particular, the initial positive (P3) deflection for T2 correct/low visibility is very low amplitude, and indeed, smaller than that for the other conditions in Fig. 6, Panel A of Pincham *et al.* (2016). This does not look like a characteristic integration pattern.

Fifthly, a reason for believing that perceptual integration is unlikely to explain our findings is that it seems T1 is immune to the decoupling of report accuracy and subjective visibility. Specifically, a second study was reported in Jones *et al.* (2020), in which we collected subjective visibility ratings for both T1 and T2. As a result, we were able to provide evidence that there is not a dissociation of report accuracy and subjective visibility for T1, i.e. Jones *et al.* (2020) failed to find an interaction between Report Measure (report accuracy vs subjective visibility) and Lag (and also identified a monotonic state-trace pattern for T1, which also suggests that report accuracy and subjective visibility were coupled for T1 at Lag 1).

This T1 immunity to the report accuracy–visibility dissociation stands against a perceptual/event integration interpretation. This is because, at its very heart, event integration suggests that a composite of T1 and T2 is experienced. However, if that were the case, one would surely expect any impairment in T2 visibility associated with that composite to also impact T1. In other words, ‘if one is going to argue that T2 subjective visibility being low at Lag 1 is due to a confused “joint” binding, why would that decoupling of subjective visibility and report accuracy not also impact T1’?

Additionally, of course, the immunity of T1 to the report accuracy–subjective visibility dissociation suggests that the relationship between working memory encoding and conscious perception is unchanged across lags and, notably, that co-activation of T1 with T2 (as it occurs at very short lags) does not impair the conscious experience of T1, in the way it does T2. This finding is wholly consistent with the serial experience interpretation we are arguing for in this paper. That is, at very short lags, particularly Lag 1, T1 typically starts being perceived before T2 does, conferring it occupancy of the exclusive ‘focus of conscious experience’, and the, late coming, T2 is excluded. This manifests in a, relative (to report accuracy), loss of visibility for T2 but not for T1, which is what we observe. In other words, the T1 claims ‘the brain’s experiencer’ before T2 arrives and holds it until T2 has decayed, but there is no such exclusivity to the encoding into working memory.

Overall, this set of arguments would seem to counter the possibility that an integrated percept at Lag 1 explains our findings and also stand against the obvious analogue of the response-bias explanation of blindsight.

### Experience at Lag 1

Another study that informs the nature of experience at Lag 1 is Recht *et al.* (2019). They assessed both report accuracy and confidence across lags of an AB paradigm. They found that confidence tracked report accuracy at all lags apart from Lag 1 (and perhaps also at Lag 2), with confidence as low at Lag 1 as at Lag 2, despite substantial Lag-1 sparing for accuracy (see Fig. 3a of Recht *et al*.). If one puts the difference in Type-2 measures (confidence vs subjective visibility) aside, these findings are fully consistent with the difference between the attentional and experiential blink highlighted in Pincham *et al.* (2016) and Jones *et al.* (2020). However, interestingly, Recht *et al*. did not fully replicate the meta-experiential blink reported here, with meta-experience larger at Lag 1 than Lag 2 (see Fig. 3c of Recht *et al*.). These differences may arise because Recht *et al.* (2019) used confidence as a type-2 measure rather than subjective visibility. Additionally, they employed a different AB paradigm: a conjunction paradigm (Botella *et al.* 2001; Chennu *et al.* 2011) in which all items presented in the stream could be reported, with targets marked by an annulus (Vul *et al.* 2008).

### Illusory percepts

A potentially interesting phenomenon associated with the meta-experiential blink can be observed by comparing the high-visibility curves in Panels a and c of Fig. 6. In particular, for high visibility, we observe, at least visually, a blink of bits (Panel a) but apparently not of accuracy (Panel c). Importantly, while in perceptually undemanding contexts high accuracy would typically co-occur with high information, the two measures can diverge. A canonical example would be illusory percepts; these are incorrect responses, suggesting low accuracy, but as long as the illusory percept consistently generates the same response (even if wrong), this could lead to high MI. That is, MI is just a measure of the systematicity of responding, not of whether that responding is, per se, correct. Thus, it is possible that the increase in information extracted at Lag 1 compared to Lag 3 for high visibility reflects an increase in illusory perception, which would be quite plausible, since Lag-1 percepts are perhaps uniquely unusual, since two targets are being presented at almost exactly the same time (Hommel and Akyürek 2005; Simione *et al.* 2017). In further work on this issue, it would be important to rule out the possibility that this effect results from a ceiling effect for accuracy in high visibility.

### Theories of the attentional blink

A central observation made in Wyble *et al.* (2009) is that while (report accuracy) performance is high at Lag 1 (i.e. there is Lag-1 sparing), it comes at a cost. The cost discussed in that paper was a loss of episodic distinctiveness (e.g. an increase in order errors and feature misbindings). This observation of a cost at Lag 1 is pivotal to the line of argument that the AB is a functionally useful mechanism, which (at Lags 2, 3 and 4) prevents episodic errors (Wyble *et al.* 2009, 2011). Indeed, this argument underlies the STST explanation of the AB.

The demonstration of an experiential blink (Pincham *et al.* 2016) suggests that there is a further cost at Lag 1, but it is experiential—the T2 has low visibility. The identification of a meta-experiential blink takes this even further, by suggesting that there is an even further cost, but this is meta-experiential—visibility is decoupled from objective report. Furthermore, the meta-experiential cost at Lag 1 seems to be even more severe than the experiential cost—compare the decline from Lag 2 to Lag 1 in the green curves and purple curve in Fig. 7.

This all provides support for the basic position that there is a substantial cost at Lag 1, not only episodic but also subjective and, indeed, meta-subjective. This provides further support for the principles underlying the STST theory of the AB (Bowman and Wyble 2007).

### Readout-enhanced STST

Despite no changes having been made to the core STST model, its readout enhancement generates a pattern of data that matches well to the human visibility data (see Fig. 8).

Another way to consider the readout mechanism added to STST and its implementation of the SESE idea is that what we are observing reflects the unity of conscious experience (Bayne 2010). That is, we can encode into working memory in parallel, since it is not subject to the same unity constraint. However, conscious percepts are in some sense serial—we have to complete one before we start the next. This is exactly the exclusivity mechanism associated with the meta-level additions of reSTST; c.f. Fig. 3.

**Figure 9.**
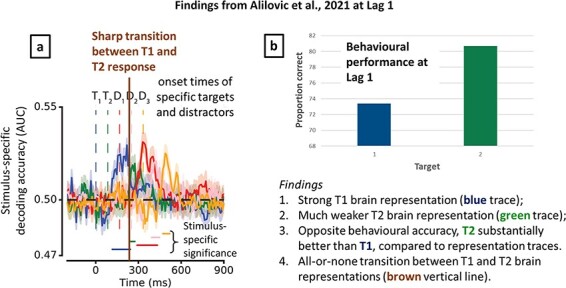
AB decoding results from Alilović *et al.* (2021), providing independent evidence for the SESE hypothesis. (a) Decoding of the T2 response (in green) is substantially weaker than the decoding of the T1 (in blue), with periods of significance indicated with coloured horizontal lines. There is a sharp transition, with no overlap of significance between T1 and T2, suggesting serial experience. (b) In striking contrast, consistent with Lag-1 sparing, T2 report accuracy is substantially higher than T1 report accuracy, suggesting simultaneous encoding. Panel a is reproduced with permission from Alilović *et al.* (2021), copyright Academic Press.

New findings reported in Alilović *et al.* (2021) give further credence to the SESE hypothesis. Alilovic *et al*. report an AB study, which through decoding is able to isolate the T1 and T2 (as well as distractor) responses at Lag 1 (in typical ERP work, the Lag-1 T1 and T2 present as a conjoint ERP response). The key comparison shown in Fig. 9a is between the decoded representation for T1 (blue trace) and T2 (green trace). If one interprets these decoding traces as indexing subjective experience, as we have argued is the case for the P3 (Pincham *et al.* 2016), then what is observed in Fig. 9a is exactly what we would expect to observe. That is, the decoded representation for T2 is much smaller than for T1, consistent with the low visibility observed for T2 at Lag 1 but not report accuracy (see Fig. 9b). Perhaps most strikingly, there is a sharp transition between the decoded representation for T1 and T2 at Lag 1, suggesting serial experience, i.e. the T2 can only start being experienced when the T1 has finished. However, further work needs to understand the relationship between decoded representations of the kind reported in Alilović *et al.* (2021) and P3s, which typically have a longer latency and also changes in amplitudes of decoding responses that seem to obtain as one progresses along an RSVP stream.

The identification of a serial experience pattern resonates with a number of previous findings. For example, Marti *et al.* (2010) provided evidence that participants’ introspections during the psychological refractory period are blind to the T2 presentation, while the T1 is being centrally processed. In other words, the T2 cannot start to be experienced until the T1 has finished.

### Postdictive effects and retro-perception

The work presented in this paper is relevant to the current debate concerning the nature of postdictive effects (Herzog *et al.* 2020); i.e. effects of later-presented stimuli on the conscious perception of earlier-presented stimuli. Sergent *et al.* (2013) and Sergent (2018) are especially interesting examples of such effects.

In respect of postdictive effects, our use of the term perceptual moment is not intended to imply a fixed-length window of integration. Indeed, the central idea of the reSTST model is that the length of integration windows is determined by the length of trains of salient stimuli in the environment. This is how the model is able to simulate the findings on ‘spreading the sparing’ in the AB (Wyble *et al.* 2009, 2011), whereby the sparing window can be extended out from Lag 1 to longer sequences of unbroken targets.

In fact, in many respects, the two-stage model of STST is similar to the informal two-stage theory of postdiction proposed by Herzog *et al.* (2020). In particular, STST incorporates an extended (across a number of 100s of milliseconds) integration period in stage one, followed by conscious perceptual episodes in stage two, which are (when episodes are sufficiently separated) associated with discrete tokens.

An important further aspect that STST emphasizes is the distinction between integrable and non-integrable stimuli presented during the perceptual moment. In RSVP, perception in the Lag-1 window seems to be dramatically different when the targets can and cannot be meaningfully integrated. In the former case, Akyurek and co-workers have run a range of experiments, with two targets presented at Lag 1 that can be combined into another potential target, e.g. Hommel and Akyürek (2005). In this case, the brain tends to experience the integrated percept and does so vividly (Simione *et al.* 2017). In contrast, in the latter case, which would correspond to a standard Lag-1 presentation, as we have shown here, the T2 is strongly encoded but poorly experienced.

Amongst postdictive effects, it is especially interesting to consider how the STST framework would explain retro-cueing of perception (Sergent *et al.* 2013), i.e. the capacity to induce perception of a previously presented target by presenting a (retro-) cue STST exhibits visual persistence in its first stage, with the Masking layer implementing an analogue of Iconic memory and the Item layer a more persistent higher-level memory, which might be related to Fragile memory (Sligte *et al.* 2008). Thus, although not currently implemented, one could envisage a cueing mechanism that caused an enhancement of an existing (target) activation trace, propelling the corresponding representation into stage two and awareness.

Also, Sergent *et al*. failed to find any evidence for blindsight, which may seem to stand against our highlighting of the possibility of sight-blind recall. However, a difference between our experiential and meta-experiential Lag-1 effects and the retro-perception effects identified in Sergent *et al.* (2013) is that the two stimuli presented in the same ‘moment’ are both targets at Lag 1, while being a target and a (retro-) cue in retro-perception. Thus, the task being performed by the brain is somewhat different; in particular, Lag 1 requires two discriminating encodings, sufficient for identification report.

In Sergent (2018), a proposal is made for the brain’s sensory processing and conscious perception streams. This is proposed at least in part to explain postdictive and particularly Sergent’s retro-perception findings. There are many aspects of this proposal that are consistent with the STST framework. Supplementary Material 4 directly explains how each point in Sergent’s (2018) proposal is realized by the STST framework.

### Relationship to other models of self-observation

Meta-experiential self-observation is common to many models, including connectionist interpretations of consciousness (Cleeremans 2014), Bayesian observers (Fleming 2020) and the reSTST model (Jones 2020). In the case of the Bayesian HOSS (Fleming 2020), a higher-order node encodes a low-dimensional, abstract signal of the presence or absence of perceptual content in lower layers of the network. Awareness reports are then governed by second-order (metacognitive) inference about the state of a first-order (perceptual) network. Such an architecture is closely aligned with the current extension of reSTST, in which a meta level encodes a low-dimensional visibility signal ‘extracted from’ first-order content. However, as currently formulated, reSTST is not Bayesian and, as a result, does not implement a generative model of perception, as is central to HOSS.

### Relationship to global workspace

**Figure 10.**
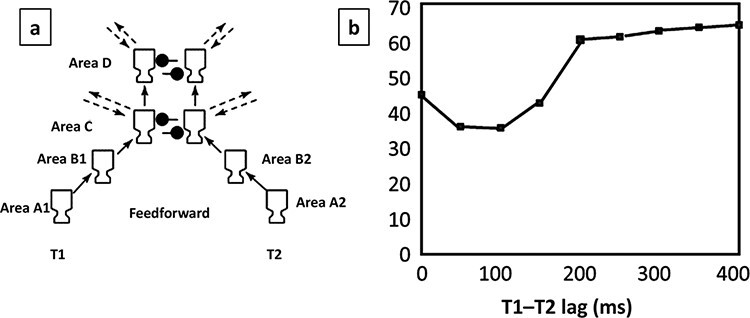
The original NGW model and simulated data. (a) Feedforward connectivity of model shown as extrinsic connectivity between cortical columns. The mechanism that generates the AB is the lateral inhibition (connections with filled black circles) between T1 and T2 cortical columns in Areas c and d. (b) Blink curve generated by original NGW model. *Y*-axis is T2 correct. Reprinted from Dehaene *et al.* (2003), with permission, copyright (2003) National Academy of Sciences, U.S.A.

The global workspace is a key theory of conscious perception. The original neuronal global workspace (NGW) model was presented as a theoretical explanation of the AB (Dehaene *et al.* 2003), which was based upon a direct competition between T1 and T2. This can be seen in Fig. 10a (see Areas c and d). The STST model of Bowman and Wyble (2007) and Wyble *et al.* (2009) stands in contrast to the NGW. This is the ‘simultaneous’ in Simultaneous Type. That is, the original STST model does not assume a fundamental representational constraint meaning that only one target can be represented at a time, with competition between T1 and T2 employed to enforce such an exclusivity constraint. Rather, STST interprets the AB as a functionally ‘deliberate’ mechanism, whereby the T2 is ‘held-out’ (by withholding attentional enhancement), while the T1 is encoded into working memory (Bowman and Wyble 2007; Bowman *et al.* 2008), and this is done specifically to prevent a confused agglomerated encoding of T1 and T2 together. Thus, as discussed earlier, the AB is viewed as an adaptive mechanism that ensures the ‘episodic distinctiveness’ of encoding into working memory, in particular that T1 is encoded as a distinct item.

The difference between these two interpretations—competition (capacity-limited) vs preserving episodic distinctiveness—really bites at Lag 1. This is because report accuracy at Lag 1 suggests that T1 and T2 can in fact be successfully encoded together, i.e. simultaneously. STST interprets this as an episodic ‘failure’ of the system, with the cost of a loss of episodic distinctiveness (e.g. high-order or conjunction errors), c.f. Sparing at a Cost (Wyble *et al.* 2009), although we can now see that there is also an experiential and meta-experiential cost.

Competitive interpretations, however, struggle with the Lag-1 data point, since it should be the point at which T1 and T2 compete the most, but why then is performance typically high on both T1 and T2? [Note that Dehaene *et al.* (2003) exhibit sparing at Lag 0 (T1 and T2 presented simultaneously)—a presentation of stimuli that is not performed in typical RSVP—and a deepest point of the blink at an SOA where sparing is normally observed; c.f. Fig. 10b].

A possible resolution of this difference between models would be that at very short lags (around a T1–T2 onset asynchrony of 100 ms) working memory encoding is simultaneous and non-competitive, in the sense of the classic STST model, but subjective visibility is more consistent with a competitive pattern of data. This is the simultaneous encoding but serial experience idea. Thus, while the STST framework models both these cases, it may be that the original NGW is conceptually a better model of experience (which is actually more consistent with its original focus) and, even, meta-experience than the classic (objective) encoding blink.

There is a further sense to which our findings may have implications for the global workspace theory of conscious perception. The dissociation we have identified here [and in Pincham *et al.* (2016) and Jones *et al.* (2020)] between report accuracy and subjective visibility at Lag 1 suggests that access and conscious experience are not synonymous and, specifically, that there can be access (recall from working memory) without conscious experience [note that phenomenological awareness would amount to conscious experience without access (Block 2007), i.e. the other direction of implication]. Indeed, access without awareness is implicit in the non-conscious working memory theory in general (Soto *et al.* 2011; Soto and Silvanto 2014; Trübutschek *et al.* 2017). Consistent with the P3 findings in Pincham *et al.* (2016), this then suggests that the brain-scale state that corresponds to the global workspace is engaged by conscious experience but is not necessary for working memory encoding and thus access.

Indeed, our findings could be seen to inform the debate between global workspace and HOT theories of conscious perception. In particular, some may argue that the SESE idea, which at short lags implies encoding of T2s without their experience, suggests that first-order representations in working memory are not enough to generate experience. Something else is required for experience, and that other thing would naturally be viewed as meta-level self-observation, as suggested by HOT theories.

### Activation-silent working memory maintenance

A decision made very early in the development of the STST theory and discussed in Bowman and Wyble (2007) was to develop an activation-based rather than synaptic change-based theory of working memory. This was because of the rapid presentation of stimuli in RSVP and the resulting requirement that encoding is rapid. We felt that such rapid encoding was unlikely to be carried by synaptic change. In particular, in RSVP, stimuli are only very briefly presented and are masked by early and later stream items. It seemed to us unlikely that such fleeting representations could induce synaptic change under e.g. Hebbian learning. This may seem to place us at odds with the ‘activity-silent’ proposals (Mongillo *et al.* 2008; Stokes 2015) that are currently prominent in WM research.

Notably, the ‘activity-silent’ theories are typically justified by experimental evidence that does not involve the fleeting (fringe of awareness) presentation of stimuli that is inherent to RSVP [however, masked stimuli were explored in Trübutschek *et al.* (2017)].

This said, it is possible that the STST perspective on WM could be reconciled with the observations underlying the activation-silent theory.

1)‘Sparse maintenance representation’: a central aspect of STST emphasized in Bowman and Wyble (2007) is that the model ‘does not employ full active maintenance’. That is, the recognition circuitry for an item is freed up once it is encoded. This is required in order for the brain to see repetitions. Indeed, STST proposes that the representation maintained is substantially reduced compared to that required for recognition; indeed, one might describe it as a sparse representation (Stokes 2015). This reduced representation is sufficient to enable the full representation to be reactivated, but it is not the full representation itself. Specifically, based upon the gate-trace circuits introduced in Bowman and Wyble (2007), the model assumes sustained activation just in a small number of inhibitory interneurons, which commit a token and a set of binding pool units. Furthermore, it may be that the contribution of inhibitory neurons to recorded activity is small, especially if these are limited in number, giving the impression of activation-silent maintenance.

2)‘Hebbian Enhancement’: it would be possible to augment the activation-based binding pool in STST with a plastic synaptic association, which might increase synaptic connections among tokens, binding pool units and types using Hebbian learning. These synaptic connections may progressively take over the representation of WM bindings as the maintenance period extends. This, however, would have consequences for the explanation of WM capacity inherent to the STST approach, which focusses on the binding pool as a capacity-limited binding resource that is ‘used up’ by items held in memory (Swan and Wyble 2014). Quite how such a capacity limit could arise from synaptic change is less apparent, where it is not completely clear what the limited resource would be. For example, long-term memory, which is classically viewed as synapse-based, is not limited to a small capacity.

More broadly, the reSTST theory presented here is largely consistent with the findings in Trübutschek *et al.* (2017), which presents an impressive magnetoencephalography (MEG) characterization of the neural correlates of non-conscious working memory. In particular, Trübutschek *et al.* (2017) provide evidence that WM (encoding and maintenance) with conscious perception exhibits similar (high-amplitude) MEG signatures to conscious perception, while WM without conscious perception exhibited very little if any MEG response. In our work, it is difficult to isolate the T2 evoked response, since a combined P3 is observed at Lag 1, but we have argued that presence–absence of the T2 P3 seems to be driven by conscious perception rather than working memory encoding (Pincham *et al.* 2016). This is exactly what one would expect from Trübutschek *et al.*’s (2017) findings.

Additionally, if we assume that the meta-level trace generated in the reSTST model is the main driver of the observed MEG response, then our model is also consistent with Trübutschek *et al.* (2017). That is, encoding without conscious perception will, as discussed in the previous paragraphs, generate sparse, potentially very difficult to image, representations, while conscious experience with associated WM encoding will generate a substantial evoked signature.

### Modelling in consciousness studies

Critical to arriving at a more theoretically informed phase of consciousness studies is providing computationally explicit theories that can be directly related to empirical findings in a detailed fashion. This requires theories to be realized in a computationally and/or mathematically precise manner, verifying those models against existing empirical evidence and providing testable predictions to ‘close the loop’ of empirical science.

One particular objective of this paper was to provide a case study in computational modelling in consciousness studies. In this respect, we have taken an existing neural network model that was originally formulated as a model of attention and working memory encoding and added a readout mechanism that enables it to model conscious experience and indeed meta-experience. Importantly, we did not adapt the parameters of the original model. We then showed that the model can, with a good deal of qualitative accuracy, fit behavioural and EEG data showing an interesting dissociation between objective performance and subjective experience. Such direct matching to observed findings is critical to the well-grounded forward progress of a field such as consciousness studies.

Additionally, in order to distinguish between different potential theories and computational models of a set of experimental findings, it is critical that predictions from computational models are fed-back to experimentalists. Furthermore, counter-intuitive predictions are especially important in this respect, since they are likely to only be true if the model making them is true—consider e.g. the empirical effort to verify general relativity by observing the position of stars during an eclipse (Coles 2001). In this spirit, we finish with the following predictions arising from the reSTST model, versions of which were also presented in Jones *et al.* (2020).

As a reflection of serial experience, the first prediction is that the P3 at Lag 1 does not have the form of a double-‘amplitude’ single-target P3. Note that the vanilla STST, without readout enhancement, does generate a double-amplitude P3 at Lag 1, see Fig. 7 of Craston *et al.* (2009). Critically, it is important to rule out the possibility that the observed Lag-1 P3 is reduced in amplitude because it is at ceiling. That is, the specific prediction is that the Lag-1 P3 is a similar amplitude to a single-target P3, and the distribution of P3s observed is not skewed according to a ceiling effect. The second prediction that the SESE P3 readout mechanism proposes is that the SSVEP weakens or even completely de-synchronizes during the P3. This is because if one asserts that an ongoing P3 for a target excludes the activation trace for another target, it should also exclude or dampen the activation traces of distractors (which drive the steady-state response). Clearly, the SSVEP is at least partially from generators substantially earlier in the processing pathway than those that might directly drive the P3. Nonetheless, some sort of reduction in the power of the SSVEP may be observable.

## Supplementary Material

niac003_Supp

## Data Availability

Data are available on request.

## Footnotes

1 Some dual-target paradigms do generate Lag-1 sparing for subjective visibility. However, the dissociation between report accuracy and subjective visibility is still observed, i.e. the interaction between measure (report accuracy vs subjective visibility) and lag is still present. Behavioural findings of this kind are extensively discussed in Jones *et al.* (2020).
