# Supplementary material for: Modelling the simultaneous encoding/serial experience theory of the perceptual moment: a blink of meta-experience
Source: Neurosci Conscious. 2022 Mar 2;2022(1):niac003. doi: 10.1093/nc/niac003 (PMC8889941; doi:10.1093/nc/niac003)
Supplement: niac003_Supp [file niac003_supp.zip › Supplementary Material.docx]

**Supplementary Material 1: Mathematical Introduction to Mutual Information**

*Discrete* mutual information quantifies the amount of information that one (discrete) probability distribution 𝑋, tells us about another (discrete) probability distribution 𝑌.

We begin with the concept of *self-information* or *information content*, which defines the amount of information gained from any particular outcome 𝑥_𝑖_ from the discrete probability distribution 𝑋. More formally, let 𝑋 be a discrete probability distribution with 𝑥_𝑖_ a result of sampling 𝑋 with 𝑃(𝑥_𝑖_) = 𝑝_𝑖_ with 0 ≤ 𝑝_𝑖_ ≤ 1, ∀ 𝑝_𝑖_, then the *self information* of 𝑥_𝑖_ is:

(𝑥_𝑖_) = − log 𝑝_𝑖_

Intuitively, the rarer the chance of 𝑥_𝑖_ occurring, the more information is conveyed by its appearance. Self-information quantifies this, in units of information. Since we are quantifying *bits* of information all logarithms are base 2. For an event with two equally likely alternatives 𝑒_𝑖_ (𝑝_𝑖_ = 0.5), (𝑥_𝑖_) = 1. This means that one *bit* of information corresponds to the ability to distinguish two equally likely alternatives.

The information transmitted from not just a single event, but across sets of events is the *entropy*. That is, the entropy of a set of events 𝑋 is the expected value of the self information, i.e.

(𝐸) = − ∑ 𝑝_𝑖_ log 𝑝_𝑖_

∀𝑖

Now, let 𝑋 and 𝑌 be discrete probability distributions with joint distribution (𝑋, 𝑌), the *mutual information* of 𝑋 and 𝑌 is defined as:

𝐼(𝑋; 𝑌) = 𝐻(𝑋) + 𝐻(𝑌) − 𝐻(𝑋, 𝑌)

That is, the amount of information carried by each distribution on its own, *less* the information that can be gathered from the joint distribution of the two on its own.

For example, consider a participant presented with one of four letters chosen uniformly at random, with the task of identifying them. We wish to calculate the information carried by the responses (R) about the stimuli (S), 𝐼(𝑆; 𝑅).There are four letters that are all equally likely to be presented, so the stimulus distribution carries 2 bits of information(∑_∀𝑖_ 𝑝_𝑖_ log 𝑝_𝑖_ = −4 × (0.25 log_2_ 0.25) = 2). If the participant correctly identifies every letter, the response and joint distributions will also carry 2 bits of information, so 𝐼(𝑆; 𝑅) = 2 + 2 − 2 = 2 bits. That is, if responses perfectly match stimuli then by knowing 2 bits of information about the responses, we know 2 bits of information about the stimuli. Consider the converse, our participant responds completely at random. In this instance, our stimulus and response distributions would still carry 2 bits of information, but this time the joint distribution will carry 4 bits of information ∑_∀𝑖_ 𝑝_𝑖_ log 𝑝_𝑖_ = −16 × (1/16 log 1/16) = 4. Mutual information will then be (𝑆; 𝑅) = 2 + 2 − 4 = 0. If responses are completely independent of stimuli, then knowing the responses gives us no information about the stimuli.


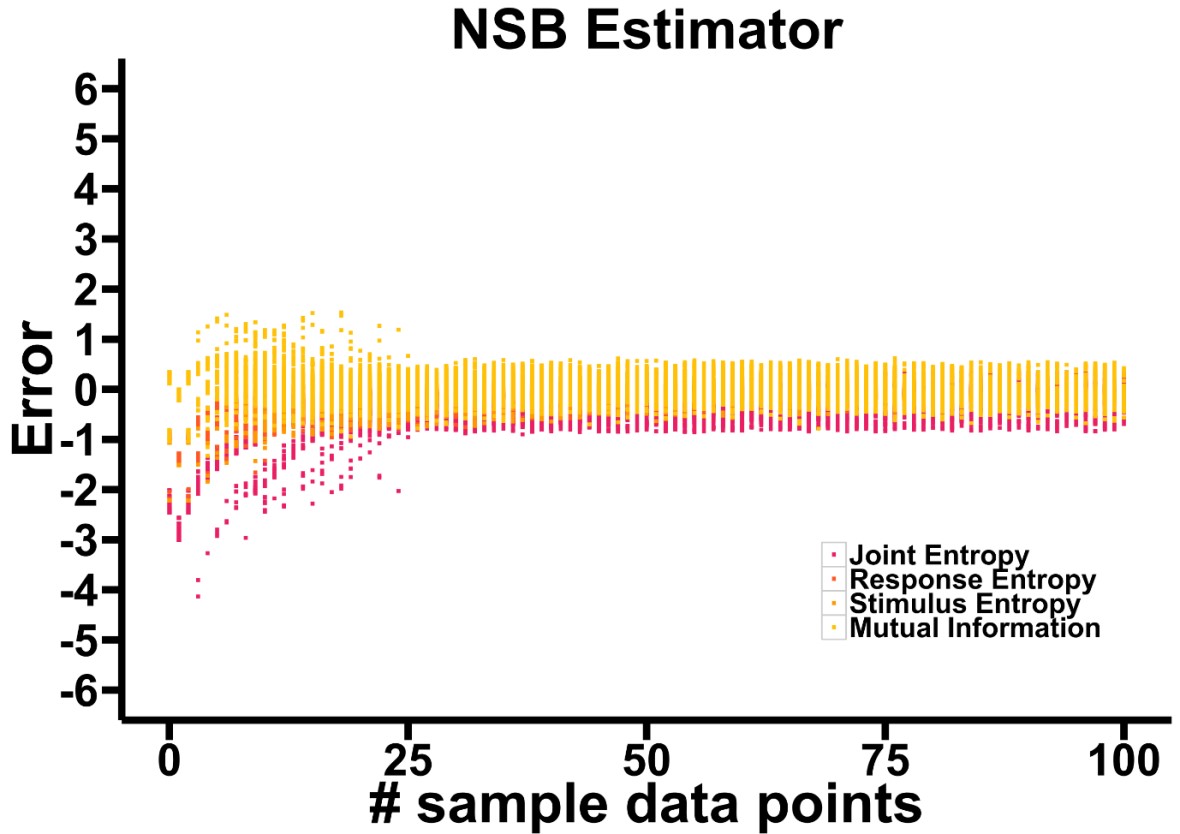


*Figure 2.1: Error of the NSB estimator of stimulus, response and joint entropy, and mutual information for a range of different sample sizes from 1 to 100. Response and stimulus entropies are effectively identical, and so stimulus entropy (orange) overlays response entropy (red) on the plot.*

**Supplementary Material 2: Mutual Information Estimation**

The application of Mutual Information is not without its challenges (Paninski 2003). In particular, entropy estimation is biased for small sample sizes. Fundamentally, with small sample sizes, entropy can be underestimated simply because the few data points available give the impression that the distribution is more concentrated than it really is. In response, we reduce the number of bins the data will be distributed across, to increase the number of data points per bin. Our merging of 6 visibility bins into 2 bins – high and low – does this. Additionally, we employ an entropy estimator. In (Jones, 2020), a set of such estimators are assessed in simulation, including the Plugin Estimator; Miller-Madow correction of the plugin estimator (Miller 1955); the JVHW estimator (Jiao, Venkat et al. 2017); the NSB (Nemenman, Shafee, Bialek) estimator estimator (Nemenman, Shafee et al. 2002) and the HS estimator (Hausser, Strimmer 2009). These simulations suggest that the NSB estimator is the most effective, and Figure 2.1 presents the results of simulations for this estimator. It can be seen that the error is close to zero once the sample size is over 20 to 25.

**Supplementary Material 3: Further Modelling Details**

As we discuss elsewhere in this paper, in order to maintain backward compatibility with the many phenomena previously modelled by STST, we have not changed the core STST model. However, since this is the first time that the model has been used to generate visibility ratings, we have flexibility with regards to how it is calculated. This supplementary material section documents this process. It builds upon the description presented in subsection “Readout-enhanced STST model” of section “Materials and Methods”.

We can highlight the following mechanisms.

1. As discussed in the Materials and Methods, an intermediate trace is only experienced if it crosses the threshold of subjectivity (and no previous stimulus is above that threshold). This parameter is denoted stre and is set to 0.0002.
2. The maximum above threshold amplitude of a T1 or T2 intermediate trace is taken as the visibility value for that target.
3. Two sources of noise are implemented, which both act on intermediate traces prior to the visibility calculation of the previous point.
   1. A simple additive noise, implemented by adding a Gaussian distributed error term (mean of zero and standard deviation of .02), is incorporated.
   2. We also include multiplicative gaussian random noise. This is included to reflect the presence of multiplicative noise in neuroimaging data; see, for example, the Fano factor (Eden & Kramer, 2010). This source of variability also avoids the somewhat counter-intuitive property of additive noise that it can (in admittedly rare cases) turn a zero-amplitude activation trace into a high amplitude, high visibility percept, i.e. induce an illusory percept. Since zero multiplied by anything is zero, such illusory percepts cannot be generated by a multiplicative source of noise. This noise has a mean of zero and a standard deviation of 1.25.
   3. We also include a function that scales this multiplicative noise by lag. This scaling is justified by the visibility response distributions shown in the supplementary material of Pincham, Bowman and Szucs (2016), in which the variability of responding with visibility ratings is high at early lags. This is a property that is consistent with the increasingly fleeting nature of the T2 percept as lag reduces. This noise scaling function is defined as follows,

$$scale\left( lag,gain \right)= e^{-(gain.\left( lag-1 \right))}$$

Thus, if the gain is set to zero, there is no scaling by lag; that is, all lags obtain the same value. As gain increases, the noise is increasingly focused on the early lags, i.e. there are greater exponential reductions as lag increases. The gain is set to 0.505 in the simulations reported here.

Finally, with regard to noise, in order to prevent values that are out of bounds, we enforce a floor of zero and ceiling of the unnoisy max. Although, this maximum is subject to a conservativeness bias in visibility responses, i.e. for participants to respond away from the theoretical maximum, according to which $adjusted\_max = max + 0.046$.

1. A nonlinear (sigmoidal) transformation of activation is applied before visibility is calculated. This has the former of an X-over-X+1 function, a standard activation function used in biologically plausible neural networks (O'reilly & Munakata, 2000). This is defined as follows,

$$y=\frac{\gamma.amp}{\gamma.amp+1}$$

where $amp$ is the input amplitude and $\gamma$ is the gain parameter. The key parameter that modulates the dynamical range of the function is the gain/steepness. As the gain approaches zero, the identity function is approached; i.e. the activation function has no effect. As the gain moves further from zero, the sigmoidal becomes steeper and more nonlinear, with a narrower dynamic range. Additionally, this nonlinear transformation is applied post enforcement of serial experience, that is, after the subjective visibility threshold is applied. This suggests a system in which a non-linear transformation occurs at the point of experience.

1. We divide into high and low visibility with respect to a (pre-noise) division point, defined as: $\theta=max/1.37$ . This places the dividing point just below ¾ of the maximum.
2. As we have always done in STST work, a retinal delay is added to the STST output. In this paper, we add the possibility that this delay may vary from trial to trial, reflecting stochasticity in the synaptic transmission from the eye. As a result, there is some temporal jitter at the single-trial level. Specifically, the single-trial T1 and T2 components are jittered by sampling latency delays from a gamma distribution and shuffling the component in time accordingly. These latencies are defined as follows,

$$lat=ceil(\sigma.\left( x-\mu\right))$$

where,

$$\sigma=8$$

$$x\sim\Gamma\left( a,b \right), a=1.5 \wedge b=2$$

$$\mu=median(\Gamma\left( a,b \right))$$

where, $\Gamma\left( a,b \right)$ is a gamma distribution, with $a$ the shape and $b$ the scale parameter. The samples taken from the gamma distribution are de-medianed, ensuring that forward and backward latency shifts are made relative to the median of the gamma distribution, i.e. a sample of the median corresponds to a zero latency, leaving the component in its original position. We also round up ($ceil$ operator) to ensure that latencies are natural numbers. $\sigma$ is a scaling parameter, larger numbers generate larger latency delays.

1. We also remove order errors generated by the model. This is because the colour-marking incorporated into the Pincham et al (2016) experiment leads to there being few order errors in the human data, with the order errors present, due to the colour-marking, not being subject to a symmetric influence between T1 and T2, as they are in the model.
2. As discussed in the Materials and Methods section, the response to distractor stimuli that is combined with target P3s to generate virtual ERPs is also suppressed by above visibility-threshold activation of T1 or T2. This is motivated by the serial experience principle at the heart of this paper. That is, the activation from distractors is reduced when a target is being experienced. We add some smoothing (by Gaussian with eight time point window) to this process, so that the suppression of distractors when targets are being experienced comes on slowly. This prevents implausible abrupt transitions in the resulting time series. We also include a down scaling (at 65%), which means that distractors are never fully suppressed. This is consistent with the SSVEP containing a contribution from early visual areas, which could be considered pre-conscious.

**Supplementary Material 4: Sergent’s offline-online theory and STST**

In Sergent 2018, a proposal is made for the brain’s sensory processing and conscious perception streams. This is proposed at least in part to explain postdictive and particularly Sergent’s retro-perception findings. There are many aspects of this proposal that are consistent with the STST framework. We highlight the points that comprise Sergent’s proposal, and link each point to an STST mechanism. Sergent’s proposal is in Italics (with verbatim quotation indicated with quotation marks) and STST mechanisms are in plain text.

*“— The stream of sensory processing is ‘online’:”*

*“(i) All incoming stimulations are processed as they arrive through fast automatic routes, with very little interference between the processing of different stimuli.”*

The simultaneous type in the title of STST, which obtains in stage-1 of the model, realises this aspect.

*“(ii) Temporal integration can occur at different scales, .. However, this remains an ‘online’ process …”*

STST integrates through time, cf. Lag-1 sparing, conjunction error simulations (Chennu et al, 2011) and the window can vary in size, cf. Spreading the sparing and Attentional Episodes (Wyble et al, 2009, 2011).

*“(iii) Ongoing brain oscillations … can modulate the strength of sensory processing.”*

STST is rate coded and thus, without oscillations. However, see Parish et al for a related oscillator-based model.

*“(iv) This unconscious stream of sensory analysis can lead to stimulus-triggered conscious perception.”*

Presenting a single stimulus to STST will initiate a cascaded sequence of processing that will lead to conscious perception.

*“(v) It can also lead to fast appropriate behaviour, when the stimulus–response association is well*

*established.”*

Although not currently implemented, over-learnt stimulus-response mappings could be maintained in the binding pool.

*“(vi) It allows fast reorienting of attention when needed, through exogenous attention.”*

This would correspond to STST’s transient attentional enhancement mechanism, called the blaster.

*“(vii) And finally, previously processed information leaves traces in the system that can be used ‘offline’ by the conscious access system.”*

A variety of different delayed conscious access patterns can arise in STST, e.g. during order errors, especially those during extended temporal episodes (Wyble et al, 2009, 2011). We are also looking to model our findings on half swaps (chapter 7 of Gootjes-Dreesbach, 2015), which have similarities to retro-perception. In particular, traces of previously processed information is left in the Item layer of stage-1.

*“— The stream of conscious access is ‘offline’:”*

*“(i) Conscious processing of one stimulus can be delayed by the processing of preceding stimuli, as is the case in PRP experiments.”*

Delayed T2 during the attentional blink is simulated by STST, and also the increased temporal variability observed during the AB (Chennu et al).

*“(ii) A previously missed event can be integrated in the stream if and when it becomes relevant, as in retroperception experiments.”*

STST would explain this through the triggering of the blaster re-invigorating a “residual” (pre-conscious) activation trace remaining in the Item/ Task Filtered layers of stage-1. This would be the mechanism by which we would explain the T1 half-order errors previously discussed (chapter 7 of Gootjes-Dreesbach, 2015).

*“(iii) Consequently, the conscious access stream can be qualified as being ‘offline’: there is no fixed delay between an external input and the moment when the representation is consciously accessed, and, furthermore, the order in which we access different representations is flexible relative to the timing of the corresponding stimuli in the external world ...”*

Order errors and conjunction errors, as modelled by STST, have this character, cf. 2f-STST model (Chennu et al, 2011). Also, our modelling of delayed and increased variability in the P3 during the blink (Chennu et al, 2009), shows “context-dependent” variability in the latency of conscious perception.

*“(iv) Importantly, this reordering should not necessarily lead to an erroneous perception of temporal order … the veridical temporal label or time-stamp of a previous input could, in principle, be reactivated, in the same way as any other aspect of the stimulus can be reactivated ..”*

This is not how STST works: it strongly associates order with experience. So, in this sense, STST makes a strong claim enabling it to be disproved.

*“(v) Constant input from the exogenous attention system prevents excessive drift of the offline system, and allows for keeping track of important events in the environment, even when the offline system is occupied by internal representations...”*

Stage-1 of STST will continuously process stimuli, while stage-2 is occupied experiencing. For example, this is required for the stronger T2s to be seen during the blink (since they out-live the unavailability of the blaster, while weaker T2s decay too quickly to benefit in this way).

*“(vi) Once a representation has been consciously accessed, it leaves a less labile trace within sensory cortices that can be integrated again at will.”*

In STST, once experienced, a reduced representation (a token and some binding pool units) provides the stored representation. This is sufficient to regenerate a sensory representation, but it is much more compressed than that activation pattern. This regenerated representation could, in principle, be integrated again.

*“(vii) Once a representation has been consciously accessed, it leaves a trace in prefrontal cortex and it is also part of our episodic memory: even if we do not currently have access to it, we know we have it, we know where to find it. This is why, even in their latent form, working memory representations can efficiently be recovered.”*

This is exactly STST: a central principle of STST is that a reduced representation (a token and some binding pool units) provide the stored representation, and an obvious neurophysiological location for tokens is pre-frontal cortex. A key aspect of tokens is that they provide a neurophysiological place from which to regenerate a memory/ sensory representation. Thus, they are exactly a place to “find” memories in a latent form and then reactivate them. Additionally, we have argued that these stored representations are fundamental to episodic representation in the brain (Wyble et al, 2009; 2011).

*“(viii) Many different factors compete for determining what will be accessed next: bottom-up factors such as exogenous attention to salient external events or salient internal representations (emotional memory), but also top-down factors either directed at external stimuli (endogenous attention), or directed at internal representations (abstract thoughts and inner speech)...”*

Attentional effects that “bias” perception becomes task-filtering in STST, which acts on the final layer of stage-1. In particular, this is how the central executive / cognitive control directs attention.

**References**

Alilović, J., van Moorselaar, D., Graetz, M., van Gaal, S., & Slagter, H. A. (2021). Representational Dynamics Preceding Conscious Access. NeuroImage, 117789.

Ashby, F.G. and Soto, F.A., 2015. Multidimensional signal detection theory. Oxford handbook of computational and mathematical psychology, pp. 13-34.

Avilés, A., Bowman, H., & Wyble, B. (2020). On the limits of evidence accumulation of the preconscious percept. Cognition, 195, 104080.

Bates, D., Sarkar, D., Bates, M.D. and Matrix, L., 2007. The lme4 package. R package version, 2(1), pp. 74.

Bayne, T. (2010). The unity of consciousness. Oxford University Press.

Block, N. (2007). Consciousness, accessibility, and the mesh between psychology and neuroscience. Behavioral and brain sciences, 30(5-6), 481-499.

Botella, J., Barriopedro, M., & Suero, M. (2001). A model of the formation of illusory conjunctions in the time domain. Journal of Experimental Psychology: Human Perception and Performance, 27(6), 1452.

Bowman, H., & Wyble, B. (2007). The simultaneous type, serial token model of temporal attention and working memory. *Psychological review*, *114*(1), 38.

Bowman, H., Wyble, B., Chennu, S., & Craston, P. (2008). A reciprocal relationship between bottom-up trace strength and the attentional blink bottleneck: Relating the LC–NE and ST2 models. *Brain Research*, *1202*, 25-42.

Brown, R. (2015). The HOROR theory of phenomenal consciousness. Philosophical Studies, 172(7), 1783-1794.

Brown, R., Lau, H., & LeDoux, J. E. (2019). Understanding the higher-order approach to consciousness. Trends in cognitive sciences, 23(9), 754-768.

Chennu, S., Bowman, H., & Wyble, B. (2011). Fortunate conjunctions revived: Feature binding with the 2f-ST2 model. In *Proceedings of the Annual Meeting of the Cognitive Science Society* (Vol. 33, No. 33).

Chennu, S., Craston, P., Wyble, B., & Bowman, H. (2009). Attention increases the temporal precision of conscious perception: verifying the neural-ST 2 model. *PLoS Comput Biol*, *5*(11), e1000576.

Chun, M.M. & Potter, M.C., 1995. A two-stage model for multiple target detection in rapid serial visual presentation. Journal of Experimental psychology: Human perception and performance, 21(1), pp. 109.

Cleeremans, A. (2014). Connecting conscious and unconscious processing. *Cognitive science*, *38*(6), 1286-1315.

Coles, P. (2001). Einstein, Eddington and the 1919 eclipse. arXiv preprint astro-ph/0102462.

Craston, P., Wyble, B., Chennu, S., & Bowman, H. (2009). The attentional blink reveals serial working memory encoding: Evidence from virtual and human event-related potentials. *Journal of cognitive neuroscience*, *21*(3), 550-566.

Dehaene, S., Sergent, C., & Changeux, J. P. (2003). A neuronal network model linking subjective reports and objective physiological data during conscious perception. *Proceedings of the National Academy of Sciences*, *100*(14), 8520-8525.

Dehaene, S., Kerszberg, M., & Changeux, J. P. (1998). A neuronal model of a global workspace in effortful cognitive tasks. *Proceedings of the national Academy of Sciences*, *95*(24), 14529-14534.

Dijkstra, E. W. (2001). Solution of a problem in concurrent programming control. In Pioneers and Their Contributions to Software Engineering (pp. 289-294). Springer, Berlin, Heidelberg.

Di Lollo, V., & Wilson, A. E. (1978). Iconic persistence and perceptual moment as determinants of temporal integration in vision. *Vision research*, *18*(12), 1607-1610.

Eden, U. T., & Kramer, M. A. (2010). Drawing inferences from Fano factor calculations. Journal of neuroscience methods, 190(1), 149-152.

Fleming, S. M. (2020). Awareness as inference in a higher-order state space. *Neuroscience of consciousness*, *2020*(1), niz020.

Fleming, S. M., & Lau, H. C. (2014). How to measure metacognition. Frontiers in human neuroscience, 8, 443.

Herzog, M. H., Drissi-Daoudi, L., & Doerig, A. (2020). All in good time: long-lasting postdictive effects reveal discrete perception. Trends in Cognitive Sciences.

Hommel, B., & Akyürek, E. G. (2005). Lag-1 sparing in the attentional blink: Benefits and costs of integrating two events into a single episode. The Quarterly Journal of Experimental Psychology Section A, 58(8), 1415-1433.

Jones, W. (2020). On the Possibility of Recalling without Seeing: Evidence from State-Trace Analysis of the Experiential Blink. PhD Thesis, School of Computing, University of Kent.

Jones, W., Pincham, H., Gootjes-Dreesbach, E. L., & Bowman, H. (2020). Fleeting perceptual experience and the possibility of Recalling Without Seeing. Scientific Reports, 10(1), 1-19.

Krueger, C. and Tian, L., 2004. A comparison of the general linear mixed model and repeated measures ANOVA using a dataset with multiple missing data points. Biological research for nursing, 6(2), pp. 151-157.

Lau, H. (2019). Consciousness, metacognition, & perceptual reality monitoring. PsyArXiv, DOI: 10.31234/osf.io/ckbyf.

Lau, H., & Rosenthal, D. (2011). Empirical support for higher-order theories of conscious awareness. Trends in cognitive sciences, 15(8), 365-373.

Lau, H.C. & Passingham, R.E., 2006. Relative blindsight in normal observers and the neural correlate of visual consciousness. *Proceedings of the National Academy of Sciences,* **103**(49), pp. 18763-18768.

Maniscalco, B. and Lau, H., 2012. A signal detection theoretic approach for estimating metacognitive sensitivity from confidence ratings. Consciousness and cognition, 21(1), pp. 422-430.

Marti, S., Sackur, J., Sigman, M., & Dehaene, S. (2010). Mapping introspection’s blind spot: Reconstruction of dual-task phenomenology using quantified introspection. Cognition, 115(2), 303-313.

Michel, M., & Lau, H. (2021). Is blindsight possible under signal detection theory? Comment on Phillips (2021).

Mongillo, G., Barak, O., & Tsodyks, M. (2008). Synaptic theory of working memory. Science, 319(5869), 1543-1546.

O'Reilly, R. C., & Munakata, Y. (2000). Computational explorations in cognitive neuroscience: Understanding the mind by simulating the brain. MIT press.

Paninski, L., 2003. Estimation of entropy and mutual information. Neural computation, 15(6), pp. 1191-1253.

Peters, M. A., & Lau, H. (2015). Human observers have optimal introspective access to perceptual processes even for visually masked stimuli. Elife, 4, e09651.

Phillips, I. (2021). Blindsight is qualitatively degraded conscious vision. Psychological Review, 128(3), 558.

Pincham, H.L., Bowman, H. and Szucs, D., 2016. The experiential blink: Mapping the cost of working memory encoding onto conscious perception in the attentional blink. Cortex, 81, pp. 35-49.

Raymond, J.E., Shapiro, K.L. & Arnell, K.M., 1992. Temporary suppression of visual processing in an RSVP task: An attentional blink? Journal of experimental psychology: Human perception and performance, 18(3), pp. 849.

Recht, S., Mamassian, P., & de Gardelle, V. (2019). Temporal attention causes systematic biases in visual confidence. Scientific reports, 9(1), 1-9.

Roberts, S. and Pashler, H., 2000. How persuasive is a good fit? A comment on theory testing. Psychological review, 107(2), pp. 358.

Rosenthal, D. (2005). Consciousness and mind. Clarendon Press.

Rounis, E., Maniscalco, B., Rothwell, J. C., Passingham, R. E., & Lau, H. (2010). Theta-burst transcranial magnetic stimulation to the prefrontal cortex impairs metacognitive visual awareness. Cognitive neuroscience, 1(3), 165-175.

Sergent, C. & Dehaene, S., 2004. Is consciousness a gradual phenomenon? Evidence for an all-or-none bifurcation during the attentional blink. Psychological science, 15(11), pp. 720-728.

Sergent, C., Wyart, V., Babo-Rebelo, M., Cohen, L., Naccache, L., & Tallon-Baudry, C. (2013). Cueing attention after the stimulus is gone can retrospectively trigger conscious perception. Current biology, 23(2), 150-155.

Sergent, C. (2018). The offline stream of conscious representations. Philosophical Transactions of the Royal Society B: Biological Sciences, 373(1755), 20170349.

Seth, A. K. (2008). Post-decision wagering measures metacognitive content, not sensory consciousness. Consciousness and cognition, 17(3), 981-983

Simione, L., Akyurek, E.G., Vastola, V., Raffone, A. & Bowman, H., 2017. Illusions of integration are subjectively impenetrable: Phenomenological experience of Lag 1 percepts during dual-target RSVP. Consciousness and cognition, 51, pp. 181-192.

Sligte, I. G., Scholte, H. S., & Lamme, V. A. (2008). Are there multiple visual short-term memory stores?. PLOS one, 3(2), e1699.

Soto, D., & Silvanto, J. (2014). Reappraising the relationship between working memory and conscious awareness. *Trends in cognitive sciences*, *18*(10), 520-525.

Soto, D., Mäntylä, T., & Silvanto, J. (2011). Working memory without consciousness. *Current Biology*, *21*(22), R912-R913.

Stokes, M. G. (2015). ‘Activity-silent’working memory in prefrontal cortex: a dynamic coding framework. Trends in cognitive sciences, 19(7), 394-405.

Swan, G., & Wyble, B. (2014). The binding pool: A model of shared neural resources for distinct items in visual working memory. Attention, Perception, & Psychophysics, 76(7), 2136-2157.

Thomas, R.D., 1999. Assessing sensitivity in a multidimensional space: Some problems and a definition of a generald′. Psychonomic bulletin & review, 6(2), pp. 224-238.

Trübutschek, D., Marti, S., Ojeda, A., King, J. R., Mi, Y., Tsodyks, M., & Dehaene, S. (2017). A theory of working memory without consciousness or sustained activity. *Elife*, *6*, e23871.

Trübutschek, D., Marti, S., Ueberschär, H., & Dehaene, S. (2019). Probing the limits of activity-silent non-conscious working memory. *Proceedings of the National Academy of Sciences*, *116*(28), 14358-14367.

Vul, E., Nieuwenstein, M., & Kanwisher, N. (2008). Temporal selection is suppressed, delayed, and diffused during the attentional blink. Psychological Science, 19(1), 55-61.

Wyble, B., Potter, M. C., Bowman, H., & Nieuwenstein, M. (2011). Attentional episodes in visual perception. *Journal of Experimental Psychology: General*, *140*(3), 488.

Wyble, B., Bowman, H., & Nieuwenstein, M. (2009). The attentional blink provides episodic distinctiveness: sparing at a cost. *Journal of experimental psychology: Human perception and performance*, *35*(3), 787.
